# The oldest known digestive system consisting of both paired digestive glands and a crop from exceptionally preserved trilobites of the Guanshan Biota (Early Cambrian, China)

**DOI:** 10.1371/journal.pone.0184982

**Published:** 2017-09-21

**Authors:** Melanie J. Hopkins, Feiyang Chen, Shixue Hu, Zhifei Zhang

**Affiliations:** 1 Division of Paleontology, American Museum of Natural History, New York, New York, United States of America; 2 Early Life Institute, State Key Laboratory of Continental Dynamics and Department of Geology, Northwest University, Xi’an, China; 3 Chengdu Institute of Geology and Mineral Resources, Chengdu, China; Naturhistoriska riksmuseet, SWEDEN

## Abstract

The early Cambrian Guanshan biota of eastern Yunnan, China, contains exceptionally preserved animals and algae. Most diverse and abundant are the arthropods, of which there are at least 11 species of trilobites represented by numerous specimens. Many trilobite specimens show soft-body preservation via iron oxide pseudomorphs of pyrite replacement. Here we describe digestive structures from two species of trilobite, *Palaeolenus lantenoisi* and *Redlichia mansuyi*. Multiple specimens of both species contain the preserved remains of an expanded stomach region (a “crop”) under the glabella, a structure which has not been observed in trilobites this old, despite numerous examples of trilobite gut traces from other Cambrian Lagerstätten. In addition, at least one specimen of *Palaeolenus lantenoisi* shows the preservation of an unusual combination of digestive structures: a crop and paired digestive glands along the alimentary tract. This combination of digestive structures has also never been observed in trilobites this old, and is rare in general, with prior evidence of it from one juvenile trilobite specimen from the late Cambrian Orsten fauna of Sweden and possibly one adult trilobite specimen from the Early Ordovician Fezouata Lagerstätte. The variation in the fidelity of preservation of digestive structures within and across different Lagerstätten may be due to variation in the type, quality, and point of digestion of food among specimens in addition to differences in mode of preservation. The presence and combination of these digestive features in the Guanshan trilobites contradicts current models of how the trilobite digestive system was structured and evolved over time. Most notably, the crop is not a derived structure as previously proposed, although it is possible that the relative size of the crop increased over the evolutionary history of the clade.

## Introduction

Although the trilobite fossil record is rich in terms of diversity and abundance, most species are known only from certain heavily mineralized exoskeletal sclerites. Non-mineralized parts, including appendages and organ systems, are preserved only under exceptional conditions, and thus are known for only a very small fraction of species. For example, traces of the digestive system are known for no more than 42 species (Table 1) of the almost 20,000 species known to date [1]. For most of these species, reconstruction of the digestive system is based only on one specimen (Table 1). The most commonly preserved structure is the alimentary canal which runs the length of the thorax along the axis. In several of these species, the alimentary canal has lobe-like features that do not extend to the genal regions of the trilobite. These have been interpreted as digestive glands, or paired metameric lateral expansions of the digestive tract (also called digestive caeca, midgut glands, or gut diverticula, but see [2] for a distinction between glands and diverticula in Cambrian lobopodians). More rarely there is evidence of an expanded region of the canal under the glabella; this has been interpreted as a stomach, or crop.

**Table 1:** Trilobite taxa for which part of the digestive system has been described.

Family	Taxon	Age	Cr	Dg	Ac	No	Ref	Figure
Ellipsocephalidae	*Bergeroniaspis lenaica*	E Camb		X	X	1	[3]	Fig 10, Plate 25, fig 1a
Nevadiidae	*Buenellus higginsi*	E Camb		X	X	Mul	[4]	Figs 2–3
Redlichiidae	*Eoredlichia intermedia*	E Camb	?	X	X	Mul	[5]	Figs 14–15
							[6]	Figs 4E, 7A, 7E
							[7]	Fig 5
Redlichiidae	*Kuanyangia bella*	E Camb	?	X	X	1	[8]	Fig 65
Oryctocephalidae	*Metabalangia yupingensis*	E Camb	X?			1	[9]	Plate 3, fig 6
Corynexochidae	*Olenoides paraptus*	E Camb	?	X	X	2	[10]	Plate 13, fig 3
							[11]	Fig 1A
Oryctocephalidae	*Oryctocephalus indicus*	E Camb			X	1	[9]	Plate 3, fig 4
Yunnanocephalidae	*Yunnanocephalus? Sp*.	E Camb		X		1	[12]	Fig 7D
Crepicephalidae	*Coosella kieri*	M Camb		X	?	1	[13]	Figs 1F, 3H
Alokisotcaridae	*Elrathia kingii*	M Camb	X		?	2	[14]	Figs 2–3
Llanoaspididae	*Genevievella granulatus*	M Camb		X	X	1	[13]	Figs 1G, 3I-M, 3O
Ptychopariidae	*Jiumenia anhuiensis*?	M Camb			X	1	[7]	Fig 2
Undetermined	*Meniscopsia beebei*	M Camb		X	X	Mul	[13]	Figs 1A-E, 3A-G, 3N
Corynexochidae	*Olenoides serratus*	M Camb		?	X	1	[15]	text-fig 4, Plate 19
Ptychopariidae	*Ptychoparia striata*	M Camb		X	X?	2	[16]	Fig 1M
							[17]	Plate 4–5
Parabolinoididae	*Orygmaspis contracta*	L Camb		X	X	1	[18]	Fig 5
Ptercephaliidae	*Pterocephalia norfordii*	L Camb	?	X	X	Mul	[19]	Figs 1–5
Olenidae	*Sphaerophthalmus*?	L Camb	X	X	X	1	[20]	Figs 1–2
Calymenidae	*Conocoryphe sp*.	Camb		X		1	[21]	Figs 1.2, 1.3
Asaphidae	*Basilicus calzadai*	L Ord	?	?	X	3	[22]	Fig 1.2, 1.3
							[23]	Fig 4C
Asaphidae	*Birmanites ingens*	L Ord	?		X	1	[23]	Fig 4C
Dalmanitidae	*Dalmanitina socialis*	L Ord			X	1	[24]	Plate III, fig 14
Trinucleidae	*Deanaspis goldfussi*	L Ord	X		X	Mul	[24]	Plate I-IV
Calymenidae	*Flexicalymene pragensis*	L Ord			?	1	[25]	Fig 5
Asaphidae	*Isotelus maximus*	L Ord			X	1	[26]	Fig 1B, 1A?
Odontopleuridae	*Selenopeltis buchi*	L Ord		X		1	[23]	Fig 3
Asaphidae	*Megistaspis (Ekeraspis) hammondi*	L Ord	X?	X	X	1	[27]	Fig 1b-d
Olenidae	*Triarthrus eatoni*	L Ord	?		X	?	[28, 29]	
Cheiruridae	*Ceraurus pleurexanthemus*	M Ord			X	1	[30]	Plate 78
Calymenidae	*Colpocoryphe bohemica*	M Ord			X	1	[25]	Fig 4
Calymenidae	*Colpocoryphe cf*. *bohemica*	M Ord		X	X	1	[25]	Fig 3
Calymenidae	*Flexicalymene senaria*	M Ord			?	1*	[31]	p. 80
Calymenidae	*Flexicalymene senaria*	M Ord			?	Dr*	[32]	Plate 4, fig 6; Plate 6, fig 2
Illaenidae	*Illaenus crassicauda*	M Ord			X	Dr	[33]	Plate 1, fig 12
Pliomeridae	*Placoparia cambriensis*	M Ord			X	1	[34]	Fig 1–4, 6
Tropidocoryphidae	*Cornuproetus cornutus*	M Dev	?			1	[14]	Fig 5A
Homalonotidae	*Wenndorfia mutabilis*	U Dev	X		X	1	[35]	Fig 1
Acastidae	*Asteropyge sp*.	Dev			X	?	[29, 36]	Plates 22–23
Phacopidae	*Phacops sp*.	Dev			X	?	[29, 36]	Plates 16–21
Redlichiidae	*Redlichia mai*	E Camb			X	2	[37]	Plates 113–114
Redlichiidae	*Redlichia noetligi*	E Camb	?			1	[37]	Plate 108
Palaeolenidae	*Palaeolenus lantenoisi*	E Camb	X	X	X	Mul	[37]	Plate 1, 123
	* *						This study	Figs 1–3
Redlichiidae	*Redlichia mansuyi*	E Camb	X			Mul	[37]	Plate 44, 45, 112
	* *						This study	Fig 1, 3, 4

Cr = crop; Dg = digestive glands; Ac = alimentary canal; No = number of specimens. E = Early; M = Middle; L = Late; Camb = Cambrian; Ord = Ordovician; Dev = Devonian.

* = specimen is a thin section through fossil; Mul = multiple specimens; Dr = Drawing only.

**Fig 1:**
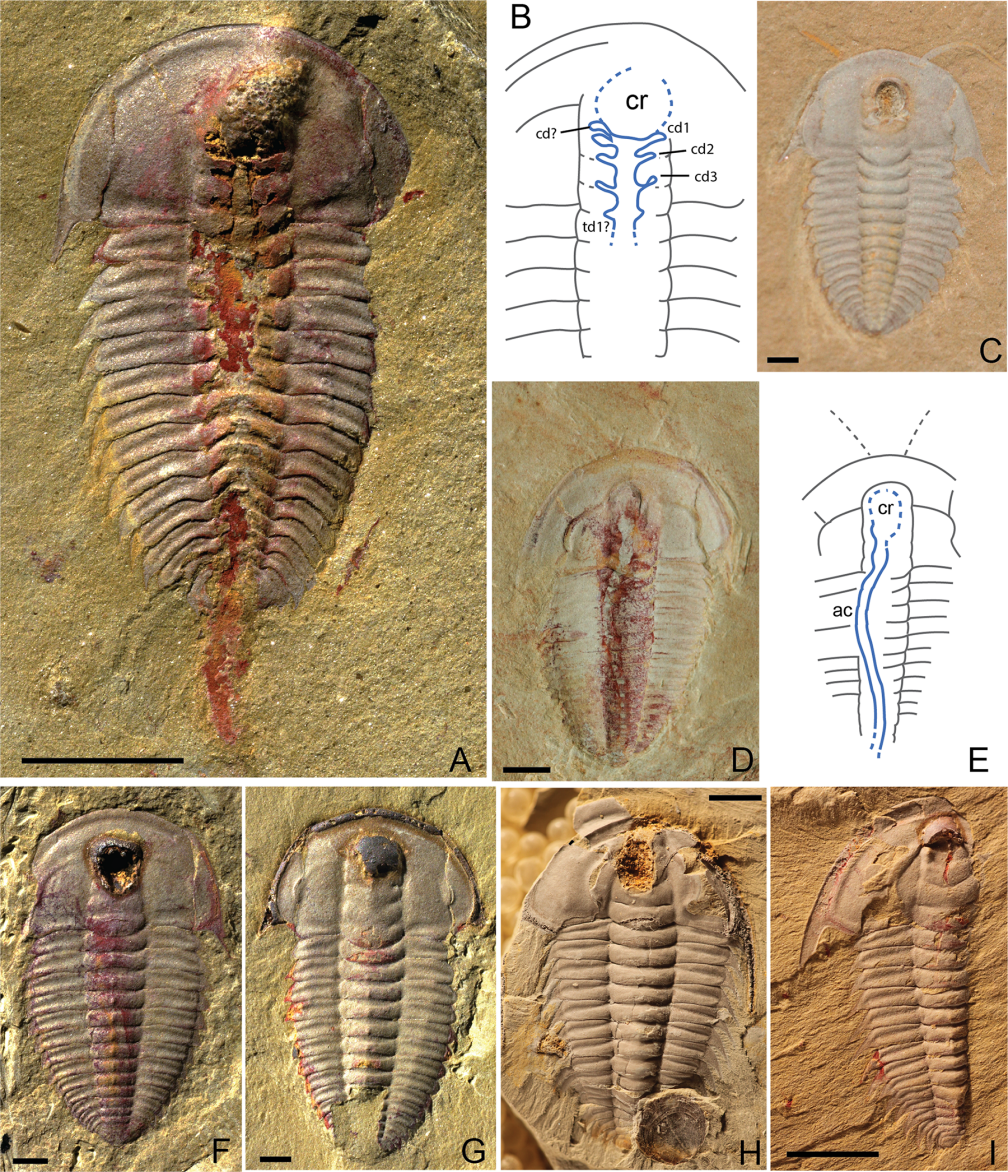
Evidence for crop and digestive glands in Guanshan trilobites. (A) *Palaeolenus lantenoisi* showing evidence of crop, digestive glands, alimentary canal along thorax, and excreted waste posterior to pygidium, GLF WLQ 228A. (B) Line drawing of (A). Cr = crop; cd = cephalic digestive glands; td = thoracic digestive glands. (C) *Palaeolenus lantenoisi*, showing crop cavity and antennae but no alimentary canal. (D) *Palaeolenus lantenoisi*, showing antennae, crop and alimentary canal but not preserving obvious digestive glands. (E) Line drawing of (D). Cr = crop; ac = alimentary canal. (F) *Palaeolenus lantenoisi*, showing crop cavity and only diffuse iron staining on thorax, GLF WLQ 174. (G) *Palaeolenus lantenoisi*, showing crop and no additional iron staining, GLF WLQ 214A. (H) *Redlichia mansuyi* showing cavity where crop would be located, GLF WLQ 216A. (I) *Redlichia mansuyi* showing crop and some additional iron staining, GLF WLQ 245A. Scale bar for (A), (H-I) = 5 mm; scale bar for all other = 1 mm.

The taxa with the most compelling evidence for a crop, such as *Deanaspis goldfussi* and *Wenndorfia mutabilis*, do not appear to have digestive glands (Table 1). As a result, it has been hypothesized that there are two main digestive systems in trilobites: one with a crop and simple alimentary canal, and one with a canal characterized by digestive glands under the cranidium (the medial sclerite of the head shield), and along at least some of the thoracic segments [29, fig 6; see also 13, 18, 23]. Thus far, there have been two possible exceptions to this pattern. The first is the remains of the digestive system attached to a juvenile hypostome (a sclerite on the ventral side of the trilobite below the cranidium) tentatively assigned to the genus *Sphaerophthalmus* [20, fig 1]. The specimen was imaged using synchrotron-radiation X-ray tomography, and as a result, the authors were able to discern delicate features, such as a J-shaped esophagus indicating that the mouth was ventrally and posteriorly directed. They also located a relatively large expanded region which they interpreted as the crop, and several pairs of lobe-like extensions on the alimentary canal which they interpreted as digestive glands. Some doubt has been raised over the latter interpretation, primarily because the lobes project ventrally rather than latero-dorsally, as in adult trilobites [23]. Even if they are digestive glands, the fact that the specimen represents a very early growth stage (early meraspid) has kept open the possibility that the crop decreased in relative size over ontogeny, in which case the two-type model based on adult morphologies would still be valid [7, 20, 23]. The second is the remains of the digestive system in an adult specimen of the trilobite *Megistaspis (Ekeraspis) hammondi* from the early Ordovician Fezouata Lagerstätte [27, fig 1b-d]. In this specimen, the putative crop is narrow, taking up only one-quarter of the width of the glabella, similar in width to the alimentary tract spanned by the gut diverticula.

The adult specimens with the most compelling evidence for a crop (Table 1), or with enlarged glabellas and muscle scars that may have supported a crop [29], are from geologically younger species (post-Cambrian). Because of this, it has been suggested that the digestive system characterized by a crop and simple alimentary canal may be more derived evolutionarily than the digestive system characterized by digestive glands [29].

Here we report on multiple specimens of two trilobite species from the Guanshan Biota (Cambrian Series 2, early stage 4) that show compelling evidence for a crop. In one species, *Redlichia mansuyi* Resser et Endo, 1937, the crop is located under the anterior region of a glabella, even though it narrows anteriorly. The other species, *Palaeolenus lantenoisi* Mansuyi, 1912, also has digestive glands. Both the presence of the crop in early trilobites and the association of the crop with a narrow glabella and digestive glands contradict previous conclusions drawn about the structure of the trilobite digestive system and its evolution.

## The Guanshan biota

The Guanshan biota is a Burgess Shale-type fossil biota [38]. Soft-bodied fossils range from the *Palaeolenus* biozone to the *Megapalaeolenus* biozone of the regional Canglangpuan Stage of South China. The Guanshan biota is thus slightly younger that the celebrated Chengjiang biota, older than the Kaili fauna (Guizhou), and temporally equivalent to the Balang fauna (Guizhou), Shipai fauna (Hubei), Sinsk biota (Siberia), and Emu Bay Shale biota (south Australia) [39]. It may also be temporally equivalent to the Sirius Passet biota, but is more likely younger [4]. The biota is preserved within the Wulongqing Formation and is widely exposed in the Kunming-Wuding and Malong-Yiliang areas in eastern Yunnan, China [39]. At least 60 taxa belonging to 10 metazoan groups and algae have been identified, including 11 trilobite species [37, 39, 40]. Previous descriptions of the biota have noted the preservation of non-mineralized (“soft-body”) anatomical features of the trilobites, including gut traces, antennae, and other appendages [37, 39], but have not described them in detail. It appears that appendages and antennae are rare, especially compared to gut traces.

Burgess Shale-type exceptional preservation has been the focus of considerable research, particularly the eponymous site in southwestern Canada and the Chengjiang biota of south China [38, 41–45]. The taphonomy of the Guanshan biota is most similar to that of the Chengjiang biota [46, 47]. Both the Chengjiang and Guanshan biotas are unusual among Burgess-Shale type fossils for having limited pyritization of non-mineralized features [38], although pyrite framboidal crystals and spherical aggregates have typically been replaced with iron oxide pseudomorphs, especially in weathered specimens [46]. Pyrite precipitation usually occurs in anoxic conditions where there is a release of sulfides from decaying organic tissues [41]. However, the formation of large euhedral crystals requires some resupply of constituents, and therefore, some diffusion in and out of the system. In addition, the formation of framboids requires nucleation and is favored under more oxidizing conditions [7]. Geochemical data from the Guanshan indicates an absence of oxygen-depleted conditions during deposition, suggesting that iron enrichment occurred during late diagenesis and subsequent alteration during weathering of exposed rocks [46]. Based on this, Forchielli et al. [46] proposed that pyrite formation occurred during late diagenesis via sulfate-reducing bacterial activity. Vannier et al. [2] drew similar conclusions from study of gut traces in lobopodians of the Chengjiang biota. Dissolution of the dorsal exoskeleton may have resulted from release of sulfuric acid during oxidation of pyrite [48].

## Materials and methods

Specimens were collected from the lower Cambrian Wulongqing Formation in the Gaoloufang section near Guangwei Village in southern Kunming, the capital city of Yunnan Province of China, during a collecting campaign of 10 local people, organized by Z. Zhang during the spring of 2014 to the autumn of 2015. The fossils were recovered from a 40–50 m thick, fine-grained laminated mudstone, occasionally intercalated with thin layers of siltstone or sandstone. Abundant silt-mud couplets with normal grading are commonly observed within the mudstone layers containing the trilobite fossils. The Wulongqing Formation contains two trilobite zones: the upper part of *Palaeolenus* Zone and the lower *Megapalaeolenus* Zone. The stratigraphic level of the specimens presented here unquestionably belong to the *Palaeolenus* Zone, generally thought to correlate with the upper Botomian Stage of the early Cambrian in Siberia [49]. Details of the stratigraphy and fossil localities were provided in Hu et al.[37, 39, 50]. A total of 270 specimens of *Palaeolenus spp*. and *Redlichia spp*. were collected, mostly by cracking out manually, and then transported by truck to the Early Life Institute (ELI) in the Department of Geology, Northwest University, Xi’an. No permits were required for the described study, which complied with all relevant regulations.

The specimens are deposited in the ELI and the Department of Geology of Northwest University, Xi’an, China. They were examined and observed under an Olympus Zoom Stereo Microscope, and photographed with the photomicrographic system of the Zeiss Smart Zoom 5, with different angles of illumination for particular views when high contrast images were required. Backscatter scanning electron microscopy (BSEM) and energy dispersive X-ray spectrometry (EDS) of uncoated fossils was performed using a Quanta 450 FEG at 20.0 kV, 60 Pa and WD of 11.4 mm at the State Key Laboratory of Continental Dynamics, Northwest University, Xi’an.

## Results

Most trilobite specimens from the Wulongqing Formation are complete or partially articulated (Figure A in S1 File). In particular, specimens have articulated librigena and sometimes the hypostome is evident. Both of these features indicate that such specimens are body fossils rather than exuvia (molted exoskeletons). The mineralized exoskeleton is preserved with some relief, but is largely compressed with minor fracturing and occasional lateral distortion. Sometimes this fracturing is concentrated along suture lines, but such “gape sutures” can occur on carcasses that were exposed to physical disturbance such as the compaction seen here [51]. The original calcareous cuticle appears to have been lost during diagenesis, but surface ornamentation was retained in impressions.

The anterior part of the glabella in several of the trilobite specimens is enlarged and characterized by dark brown or red staining (Fig 1 and Fig A in S1 File), which we interpret to be the remains of an expanded stomach, or crop. In some cases, secondary loss during weathering has occurred, leaving a cavity lined with iron-oxide spherical aggregates or framboids where the crop was (Fig 1C, 1F and 1H, Fig A in S1 File; see also Figs 3 and 4). In other cases, material that occupied what is now a cavity on the part is preserved on the counterpart. Such cavities are distinct from the crushed glabellas seen in some complete exuvia [51, 52]. In both *Palaeolenus lantenoisi* and *Redlichia mansuyi*, the crop is located at the anteriormost part of the glabella but may extend as far back as S1 (Fig 1F and 1H). Because the crop is contained within the glabella, it is relatively less wide compared to the width of the cephalon in *Redlichia spp*., than in *Palaeolenus lantenoisi*, because of the anterior narrowing of the glabella in the former. In general, the crop is elliptical in shape but not always symmetrically preserved. The crop is the most frequently preserved part of the digestive system. Red staining along the axis is less frequent and usually more diffuse (Fig 1A and 1D). Occasionally the red staining along the axis extends past the posterior margin of the pygidium, possibly representing extruded gut contents forced out by depositional compaction (e.g., Fig 1A).

**Fig 2:**
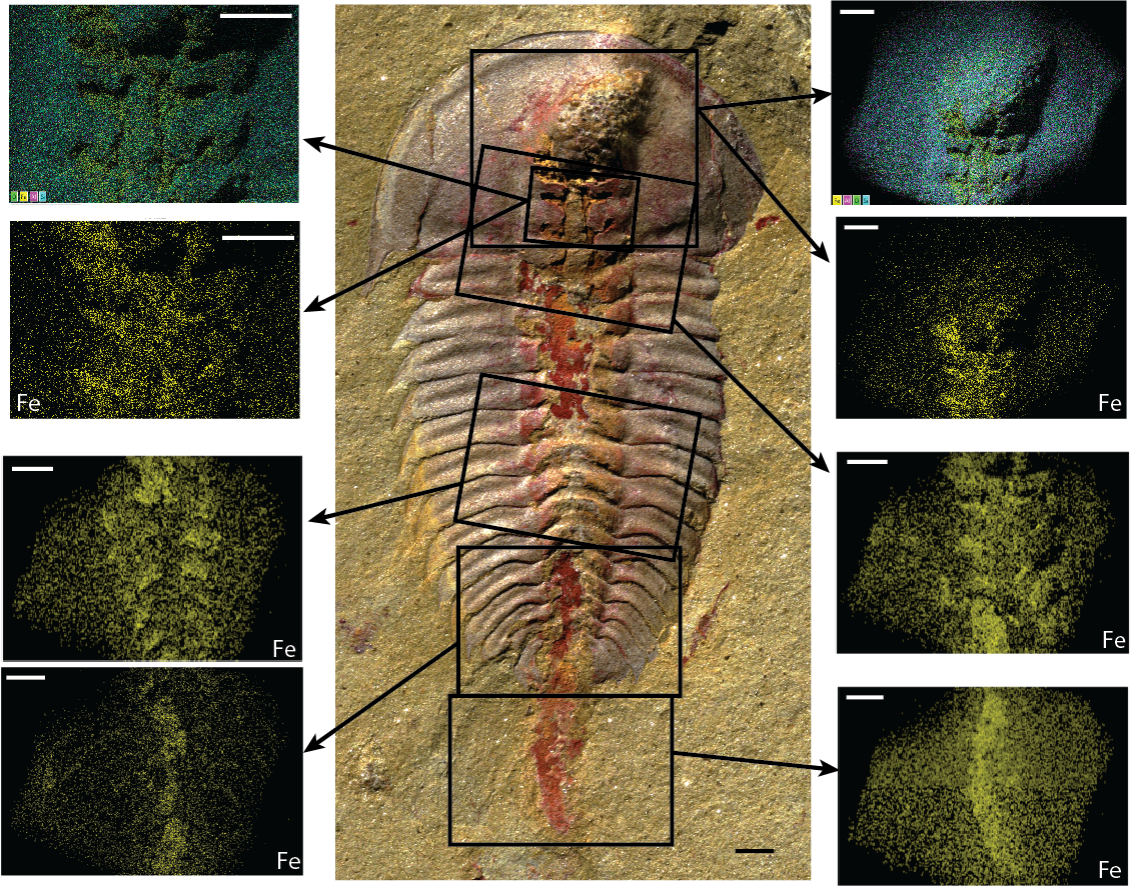
Iron concentrations in digestive tract of *Palaeolenus lantenoisi*, GLF WLQ 228A. Scale bars = 1 mm. Top right and left panels show elemental maps of Fe (yellow), Si (pink), O (green), and Al (cyan) concentrations in the areas indicated by black rectangles in the central photography. All other panels show elemental maps of just Fe concentrations (yellow) in the indicated areas. Note that here and in elemental maps in other figures, some areas where iron concentrations are expected but not evident are in ‘shadow’ due to the orientation of the specimen or because that part of the specimen is outside of the width of detection, so no elemental composition is available (compare the top two panels showing Fe, Si, O, and Al with those just below showing only Fe). See Figs B-G in S1 File for additional elemental maps of different parts of this specimen.

In one specimen of *Palaeolenus lantenoisi*, there is evidence of digestive glands just posterior to the enlarged area (Figs 1A and 2). The putative digestive glands are preserved as weathered areas with red staining, and their shape is consistent with other descriptions of digestive glands in trilobites [13, 19]: they are lobate in form, constrained within the axis, narrower than the sagittal alimentary tube, and branch dorso-laterally. They are also distributed one pair per segment; this is evident by the positioning of each pair along a glabellar furrow (cd1-3) or where the posterior margin of the occipital ring articulates with the anteriormost thoracic segment (td1). Thus they are associated with the tergite-tergite junction described by Lerosey-Aubril et al. [13] and supported by gene expression patterns in Panarthropoda [53]. We have not discovered any *Redlichia* specimens with obvious impressions of gut diverticula.

**Fig 3:**
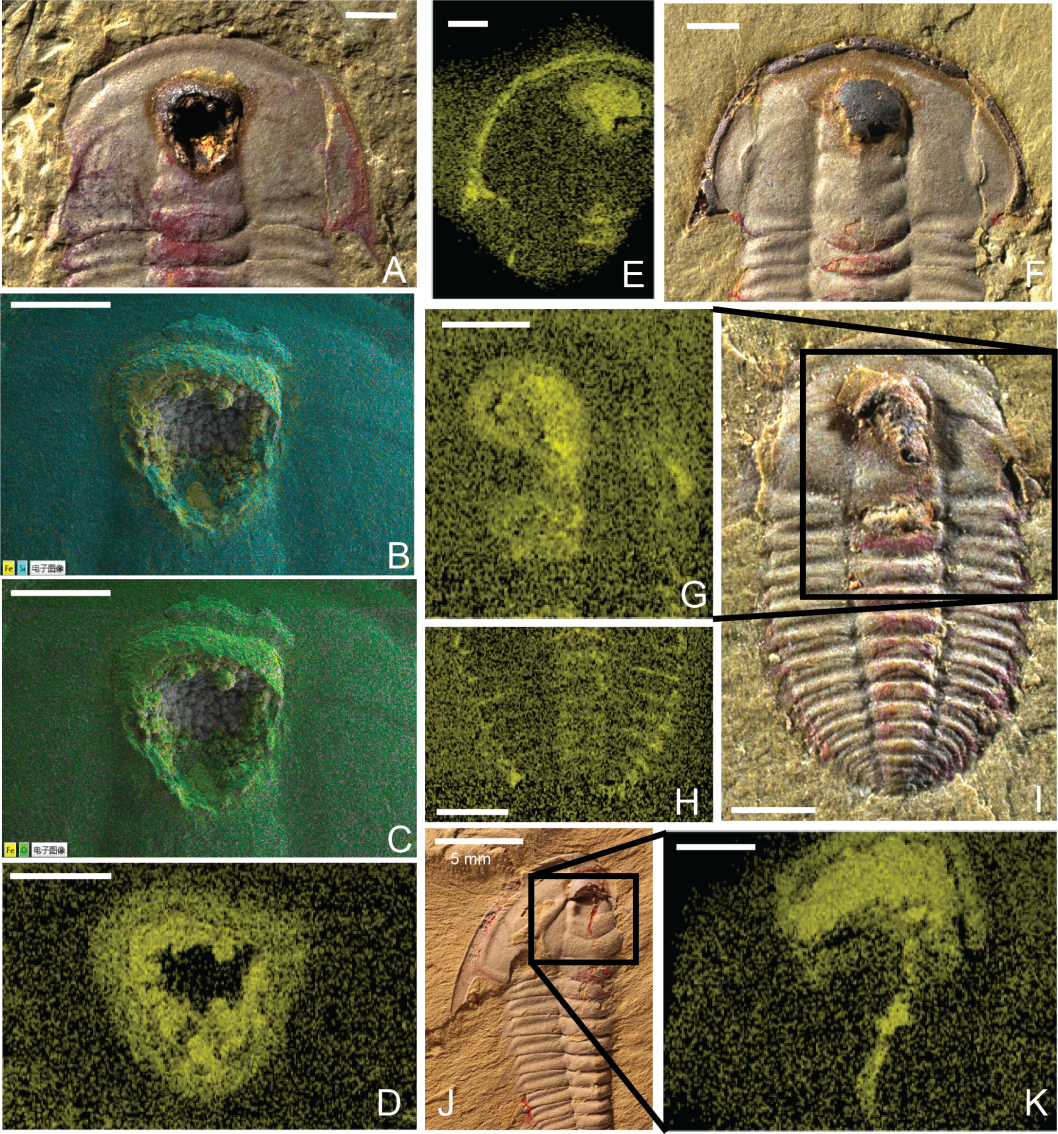
Overview of soft-body preservation. (A) *Palaeolenus lantenoisi*, GLF WLQ 174. (B) Elemental maps of Si (cyan) and Fe (yellow) overlain on SEM image of iron oxide framboids in crop of specimen shown in 3A. (C) Elemental maps of O (green) and Fe (yellow) overlain on SEM image of iron oxide framboids in crop of specimen shown in 3A. (D) Elemental map showing Fe concentration (yellow) at crop of specimen shown in 3A. There is no indication of iron in the center of the image because the framboids at the back of the cavity are outside the width of the detection field (see also 3B, 3C). (E) Elemental map showing Fe concentration (yellow) under left-hand side of doublure of cephalon, articulating half-rings on thoracic segments, and at crop for specimen shown in 3F. (F) *Palaeolenus lantenoisi*, GLF WLQ 214A. (G) Elemental map showing Fe concentration (yellow) at crop, posterior part of glabella, and palpebral lobe of specimen shown in 3I. (H) Elemental map showing Fe concentration (yellow) at thoracic and pygidial pleural spines of specimen shown in 3I. (I) *Palaeolenus latenoisi*, GLF WLQ 212A. (J) *Redlichia mansuyi*, GLF WLQ 245A. (K) Elemental map showing Fe concentration (yellow) at crop of specimen shown in 3J. Scale bar = 1 mm except where indicated. See Figs H-L in S1 File for additional elemental maps for these specimens. See Figs M-N in S1 File for additional elemental maps of pleural areas in *Redlichia spp*.

**Fig 4:**
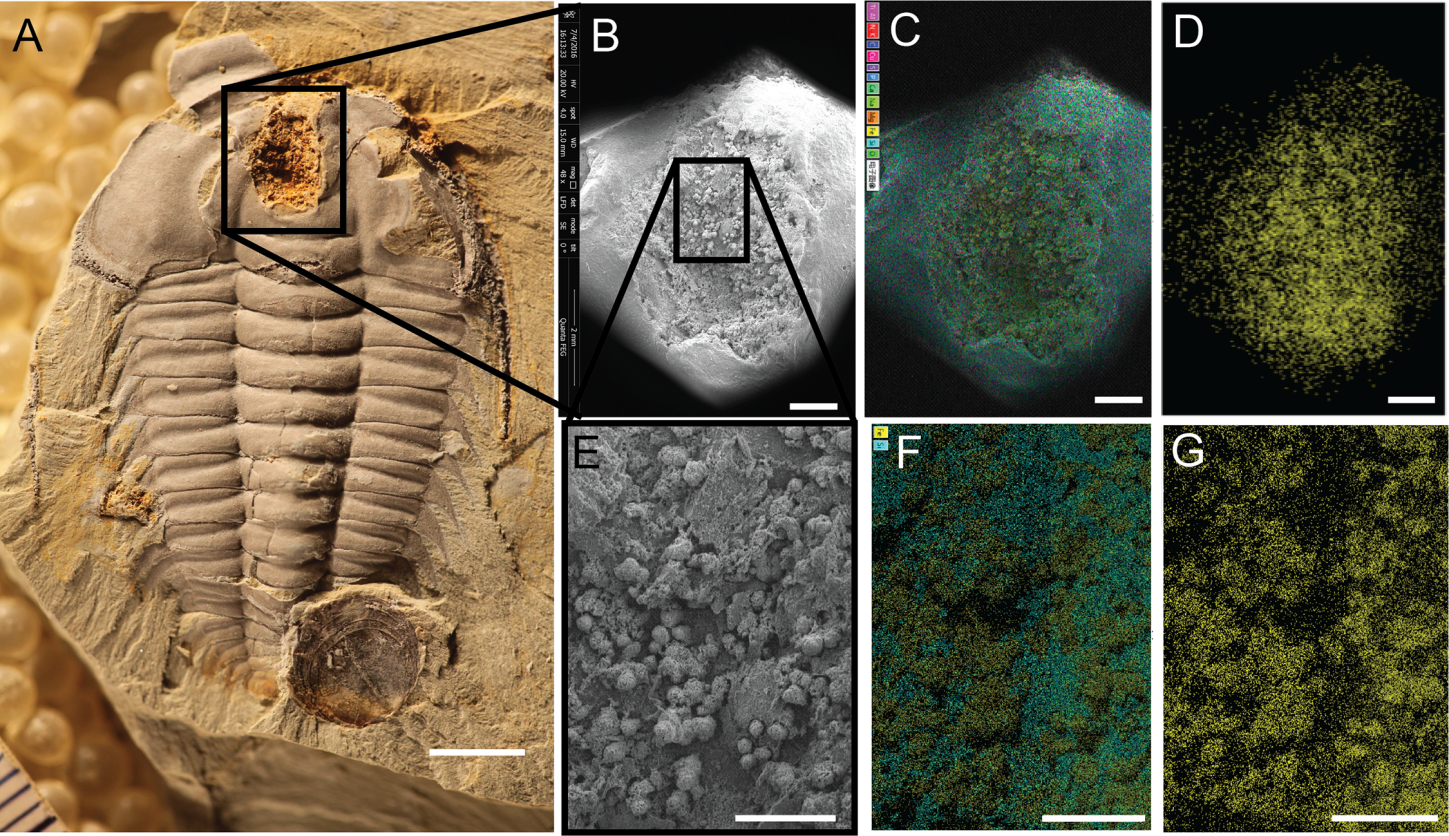
Spherical aggregates of iron oxide in crop of *Redlichia mansuyi*, GLF WLQ 216A. (A) Photograph of entire specimen. (B) SEM image of crop outlined in (A). (C) SEM image overlain by elemental mapping: Ti, Al (fuchsia); N, K (red); C (dark blue); Cu (pink); S (violet); P (light blue); Ca (blue-green); Na (yellow-green); Mg (orange); Fe (yellow); Si (cyan); O (green). (D) Elemental map of just iron in area shown in B and C. (E) SEM image of area outlined in (B). (F) SEM image overlain by elemental mapping: Fe (yellow); Si (cyan). (G) Elemental map of iron in area shown in E and F. Scale bar for (A) = 5 mm; scale bar for (B-D) = 1 mm; scale bar for (E-G) = 500 microns. See Figs O-P in S1 File for additional elemental maps for this specimen.

Elemental mapping of specimens of both *Palaeolenus lantenoisi* and *Redlichia* spp. shows that the areas with brown and red staining have high concentrations of iron (Figs 2–4). Oxygen is in high abundance and evenly distributed across the specimen and the matrix, including areas where Fe is concentrated (Figs B-P in S1 File). This indicates that the red staining is composed of iron oxides. Si, Al, and K are usually in high abundance and evenly distributed across the matrix and the impression of the exoskeleton, but frequently absent where Fe is concentrated (e.g., Fig 2, Figs L, N, and P in S1 File). Mg, Ti, C, and Ca are less abundant but generally evenly distributed (e.g., Figs B, I, and M in S1 File). S, Na, and P are only sometimes abundant in high enough quantities to be detected (e.g., Figs C, D, E, G, J and M in S1 File); W (e.g., Fig H in S1 File), Cu (e.g., Figs J and M in S1 File), and N (Fig O in S1 File) are rare.

Iron oxide concentrations are usually highest in the anterior part of the glabella, where the crop would have been (Fig 3B–3E, 3G and 3K). Iron oxide is also frequently concentrated along the cephalic margin (Fig 3E), the thoracic and pygidial pleural spines (Fig 3H), and at the articulating half-rings of the thorax (Fig 3E and 3H). These three areas of the exoskeleton were characterized by a tight fold called the doublure; organic material may have been more protected against decay in these areas.

Spherical aggregates of iron oxides are most frequently preserved in the crop cavities (Fig 4); these aggregates are in the same size range as those reported from other Guanshan fossils [46]. Occasionally iron oxide framboids are present (Fig 3B and 3C). The spherical aggregates are comprised of O (43.9%) Fe (29.0%), Si (11.9%), Al (6.1%) (Fig 4 and Fig Q in S1 File).

Of the 270 specimens examined, 118 belong to *Palaeolenus*, and 152 belong to *Redlichia*. 21 of the *Palaeolenus* specimens contained some evidence of soft-body preservation, and 19 of these contained evidence of gut traces specifically. 24 of the *Redlichia* specimens contained some evidence of soft-body preservation, but only 8 of these contained evidence of gut traces specifically.

## Discussion

The specimens examined herein have all been weathered (compare Figs 1–4 with Forchielli et al. [46], fig 2). The matrix and exoskeletal molds show high concentrations of Si, K, and Al. In contrast, Fe is sparce in the matrix and molds, but is the primary component of the gut traces, along with O. This distribution of elements has been seen in other trilobite specimens preserving gut traces. For example, the molds of trilobite specimens from the Kaili biota are composed of clay minerals with iron oxides [11], and pyrite formation and iron oxides are associated with preservation of the stomach in *Elrathia* [14]. Trilobites of the Chengjiang biota show both iron staining at digestive glands as well as the cephalic margin and pleural spines [e.g., ref 12, fig 7D], but pyrite habit in the Chengjiang biota varies in association with variation in the decay susceptibility of the soft tissues [48]. Even though the presence of iron is more diffuse in Guanshan fossils than in Chengjiang fossils [46], iron concentrations in trilobites of the Guanshan biota appear to be similar in distribution and habit.

In the Guanshan trilobites, most of the topographical resistance to compaction occurred where there was soft-body preservation. Most notably, the crop retains dimensionality, even though the exoskeleton surrounding it was flattened. This characteristic is consistent with specimens from the Burgess Shale, even though the type of mineral replacement was different [42]. Given current understanding of pyrite formation in the Guanshan and Chengjiang deposits [46], however, the three-dimensional preservation of some parts of the gut is more likely due to selective syndiagenetic anaerobic microbial decay of the internal issues or organs [54] than to early pre-compaction mineralization [42, 43]. In the former scenario, gaseous accumulations from decay could have helped to prevent compaction of the gut tissues.

Mineral infilling of some trilobite gut traces has led authors to suggest that the gut was fluid-filled [4, 54], and even that trilobites might have liquefied the prey as some arachnids do [4]. Although the latter remains speculative, there does seem to be a lack of convincing evidence that trilobites consumed large quantities of sediment as deposit feeders. For example, Al, Si, and Mg concentrations that have been reported from trilobite gut traces, and would be indicative of internal sediment processing, may actually reflect the matrix composition below the gut trace [11]. Although it is likely that trilobites were at least somewhat selective in what they consumed, interpretations of feeding behavior and trophic level based on the morphology and elemental composition of preserved gut traces remain controversial.

The Guanshan material is the first convincing evidence for a crop in early Cambrian trilobites. The frequency with which the crop is represented by an iron-oxide lined cavity provides a new search image for this part of the digestive system in fossil specimens. Only once before has a cavity been interpreted as a crop [*Deanaspis goldfussi*, 24]. Fatka et al. [23] note a similar depression in the anterior part of the glabella of *Birmanites ingens*, but conclude that while this cavity represents space enclosed by the hypostome, it cannot represent a crop because there is no obvious preservation of the digestive tract between this space and where it is visible in the thorax. However, differential preservation of the gut trace within individual specimens is not uncommon (see below). Furthermore, it is certain that the digestive system did continue under the cranidium to the mouth, even if it is not preserved. Thus, whether *Birmanites ingens* had a crop or not remains an open question.

Similarly, it is also possible that the cavities in the anterior part of the glabella in three specimens of *Basilicus calzadai* from the Izegguirene Formation of Morocco represent the remains of the crop in this species [22, 23]. In addition one specimen of *Basilicus calzadai* shows a pair of small circular cavities on either side of the alimentary canal at the anteriormost axial segment, and another fainter pair within the glabella [22, fig 1.2]. Although preservation is not good enough to be conclusive, *Basilicus calzadai* may be another example of a trilobite with both crop and digestive glands. As such, there is now evidence for this digestive system in at least four species spanning the Cambrian (*Palaeolenus lantenoisi* and *Sphaeropthalmus*? *sp*.) and Ordovician (*Basilicus calzadai* and *Megistaspis (Ekeraspis) hammondi*).

Gutiérrez-Marco and colleagues [27] interpreted the combination of a crop and digestive glands in *Megistaspis (Ekeraspis) hammondi* to be a new “type 3” trilobite digestive system. However, we suspect that at least some previous classifications may have been misled by taphonomic variabililty. In addition to the relatively small number of specimens (16% of *Palaeolenus* specimens and 5% of *Redlichia* specimens) that show gut trace preservation in the Guanshan material, there is considerable variation in the fidelity of gut trace preservation among specimens, despite having been collected from the same outcrop (Figs 1–4, Fig A in S1 File). Inconsistent preservation was also observed among specimens of *Buenellus ingens* from Sirius Passet [4]. Lin [11] went so far as to suggest that when the alimentary canal was preserved in *Olenoides paraptus*, paired axial markings were not visible, and vice versa; in other words, one feature was always preserved at the exclusion of the other. In individual specimens, it is possible that different concentrations of sediment and organic material along the digestive tract could result in differential preservation of different regions [6, 7, 23]. Thus even under the same taphonomic conditions, differential preservation would be a natural consequence of individual variation in the type and quantity of food, as well as the point in digestion of that food at death and burial. This emphasizes the importance of multiple specimens for describing the digestive system of a species [13].

The preservation of a crop in two early Cambrian Stage 4 trilobites (of different taxonomic orders) indicates that the presence of the crop is not only a feature of geologically younger trilobites, as previously suggested [29]. However, the crops in *Palaeolenus lantenoisi* and *Redlichia* are no larger than the anterior part of the glabella, which is relatively narrow in both species, and even narrows anteriorly in *Redlichia*. Taxa with expanded glabella could have accommodated a much larger crop (e.g., *Phacops*), in which case there may still be evolutionary trends toward relatively larger crops over time [29].

## Supporting information

S1 File Supporting figures A-Q.
(PDF)

## References

[1] Adrain JM. Class Trilobita Walch, 1771. In: Zhang Z-Q, editor. Animal biodiversity: an outline of higher-level classification and survey of taxonomic richness. Zootaxa 3148; 2011. p. 104–109. 10.11646/zootaxa.3703.1.1 26146682

[2] Vannier J, Liu J, Lerosey-Aubril R, Vinther J, Daley AC. Sophisticated digestive systems in early arthropods. Nat Commun. 2014;5:3641 doi: 10.1038/ncomms4641 24785191 10.1038/ncomms4641

[3] Ivantsov AY. New taxa of asaphid trilobites (Ptychopariida: Asaphinae) from the Ordovician of Leningrad Region. Paleontological Journal. 2000;34(4):411–418.

[4] Babcock LE, Peel JS. Palaeobiology, taphonomy, and stratigraphic significance of the trilobite *Buenellus* from the Sirius Passet Biota, Cambrian of North Greenland. Memoirs of the Association of Australasian Palaeontologists. 2007;34:401–418.

[5] Shu D-G, Geyer G, Chen L, Zhang X. Redlichiacean trilobites with preserved soft-parts from the Lower Cambrian Chengjiang fauna (South China). Beringeria. 1995;2:203–241.

[6] Hou X, Clarkson ENK, Yang J, Zhang X, Wu G, Yuan Z. Appendages of early Cambrian *Eoredlichia* (Trilobita) from the Chengjiang biota, Yunnan, China. Earth Environ Sci Trans R Soc Edinb. 2009;99:213–223.

[7] Zhu X, Lerosey-Aubril R, Esteve J. Gut content fossilization and evidence for detritus feeding habits in an enrolled trilobite from the Cambrian of China. Lethaia. 2014;47:66–76. doi: 10.1111/let.12041

[8] Chen J, Zhou G. Biology of the Chengjiang fauna. Bulletin of National Museum of Natural Science. 1997;10:11–105.

[9] Zhao Y, Maoyan ZHU, Babcock LE, Yuan J, Parsley RL, Peng J, et al Kaili Biota: a taphonomic window on diversification of metazoans from the basal Middle Cambrian: Guizhou, China. Acta Geologica Sinica. 2005;79(6):751–765.

[10] Yuan J, Zhao Y, Li Y, Huang Y-Z. Trilobite Fauna of the Kaili Formation (Uppermost Lower Cambrian—Lower Middle Cambrian) from Southeastern Guizhou, South China. Shanghai: Shanghai Science and Technology Press; 2002.

[11] Lin J-P. Preservation of the gastrointestinal system in *Olenoides* (Trilobita) from the Kaili biota (Cambrian) of Guizhou, China. Memoir of the Association of Australasian Palaeontologists. 2007;33:179–189.

[12] Zhang X-L, Han J, Zhang Z-F, Liu H-Q, Shu D-G. Reconsideration of the supposed naraoiid larva from the Early Cambrian Chengjiang Lagerstätte, South China. Palaeontology. 2003;46(3):447–465.

[13] Lerosey-Aubril R, Hegna TA, Kier C, Bonino E, Habersetzer J, Carré M. Controls on gut phosphatisation: the trilobites from the Weeks Formation Lagerstätte (Cambrian; Utah). PLOS ONE. 2012;7(3):e32934 doi: 10.1371/journal.pone.0032934 22431989 10.1371/journal.pone.0032934 PMC3303877

[14] Peteya JA. Resolving details of the nonbiomineralized anatomy of trilobites using computed tomographic imaging techniques. M. Sc. Thesis: The Ohio State University; 2013. Available from: https://etd.ohiolink.edu/pg_10?201068837326413::NO:10:P10_ETD_SUBID:3732

[15] Whittington HB. Exoskeleton, moult stage, appendage morphology, and habits of the Middle Cambrian trilobite Olenoides serratus. Palaeontology. 1980;23(1):171–204.

[16] Kordule V. Ptychopariid trilobites in the Middle Cambrian of Central Bohemia (taxonomy, biostratigraphy, synecology). Bull Geosci. 2006;81(4):277–304.

[17] Jaekel O. Über die Organisation der Trilobiten. Teil I. Zeitschrift der Deutschen Gesellschaft für Geowissenschaften. 1901;53:133–171.

[18] Lerosey-Aubril R, Paterson JR, Gibb S, Chatterton BDE. Exceptionally-preserved late Cambrian fossils from the McKay Group (British Columbia, Canada) and the evolution of tagmosis in aglaspidid arthropods. Gondwana Res. 2017;42:264–279.

[19] Chatterton BDE, Johanson Z, Sutherland G. Form of the trilobite digestive system: alimentary structures in Pterocephalia. J Paleontol. 1994;68(2):294–305.

[20] Eriksson ME, Terfelt F. Exceptionally preserved Cambrian trilobite digestive system revealed in 3D by synchrotron-radiation X-Ray tomographic microscopy. PLoS ONE. 2012;7(4):e35625 doi: 10.1371/journal.pone.0035625 22558180 10.1371/journal.pone.0035625 PMC3338417

[21] Fatka O, Szabad M, Budil P, Micka V. Position of trilobites in Cambrian ecosystem: preliminary remarks from the Barrandian Region (Czechia) In: Rabano I, Gozalo R, Garcia-Bellido D, editors. Advances in trilobite research. Madrid: Cuadernos del Museo Geominero, no. 9, Instituto Geológico y Minero de España; 2008 p. 117–122.

[22] Corbacho J. Trilobites from the Upper Ordovician of Bou Nemrou—El Kaid Errami (Morocco). Batalleria. 2011;16:16–36.

[23] Fatka O, Lerosey-Aubril R, Budil P, Rak S. Fossilized guts in trilobites from the Upper Ordovician Letna Formation (Prague Basin, Czech Republic). Bull Geosci. 2013;88(1):95–104.

[24] Snajdr M. On the digestive system of *Deanaspis goldfussi* (Barrande). Casopis Narodniho Muzea. 1987;156(1/4):8–16.

[25] Fatka O, Budil P, David M. Digestive structures in Ordovician trilobites Colpocoryphe and Flexicalymene from the Barrandian area of Czech Republic. Estonian Journal of Earth Sciences. 2015;64(4):255.

[26] English AM, Babcock LE. Feeding behaviour of two Ordovician trilobites inferred from trace fossils and non-biomineralized anatomy, Ohio and Kentucky, USA. Memoirs of the Association of Australasian Palaeontologists. 2007;34:537–544.

[27] Gutiérrez-Marco JC, García-Bellido DC, Rábano I, Sá AA. Digestive and appendicular soft-parts, with behavioural implications, in a large Ordovician trilobite from the Fezouata Lagerstätte, Morocco. Sci Rep 2017;7:39728 doi: 10.1038/srep39728 28071705 10.1038/srep39728 PMC5223178

[28] Cisne JL. Triarthrus eatoni (Trilobita): Anatomy of its exoskeletal, skeletomuscular, and digestive systems. Palaeontographica Americana 1981;9:95–142.

[29] Lerosey-Aubril R, Hegna TA, Olive S. Inferring internal anatomy from the trilobite exoskeleton: the relationship between frontal auxiliary impressions and the digestive system. Lethaia. 2011;44(2):166–184.

[30] Whiteley TE, Kloc GJ, Brett CE. Trilobites of New York: an illustrated guide Ithaca, NY: Cornell University Press; 2002.

[31] Raymond PE. The appendages, anatomy, and relationships of trilobites. Memoirs of the Connecticut Academy of Arts and Sciences. 1920;7:1–169.

[32] Walcott CD. The trilobite: new and old evidence relating to its organization. Bulletin of the Museum of Comparative Zoology, Harvard College. 1881;8:191–224.

[33] Volborth A. Über die mit glatten Rumpfgliedern versehenen russischen Trilobiten, nebst einem Anhange überdie Bewegungsorgane und über das Herz derselben. Mémoires de l'Academie Impériale des Sciences de St-Petersbourg, Série 7. 1963;6(2):1–47.

[34] Whittington HB. Anatomy of the Ordovician trilobite *Placoparia*. Philos Trans R Soc Lond B Biol Sci. 1993;339(1287):109–118.

[35] Alberti M. Ein homalonotider Trilobit mit "Weichteilerhaltung" aus dem Ober-Ems des Westerwaldes (Unterdevon; Rheinisches Schiefergebirge). Mainzer Geowissenschaft. 2009;37:23–32.

[36] Stürmer W, Bergström J. New discoveries on trilobites by X-rays. Paläontol Z. 1973;47:104–141.

[37] Hu S, Zhu M, Luo H, Steiner M, Zhao F, Li G, et al The Guanshan Biota. Kunming: Yunnan Publishing Group Co., Ltd., Yunnan Science and Technology Press; 2013.

[38] Gaines R. Burgess Shale-type preservation and its distribution in space and time. In: Laflamme M, Schiffbauer JD, Darroch SAF, editors. Reading and Writing of the Fossil Record: Preservational Pathways to Exceptional Fossilization. 20: The Paleontological Society Papers; 2014. p. 123–146.

[39] Hu S, Zhu M, Steiner M, Luo H, Zhao F, Liu Q. Biodiversity and taphonomy of the Early Cambrian Guanshan biota, eastern Yunnan. Science China Earth Sciences. 2010;53(12):1765–1773.

[40] Luo H, Li Y, Hu S, Fu X, Hou S, Liu X, et al Early Cambrian Malong Fauna and Guanshan Fauna from eastern Yunnan, China. Kunming: Yunnan Science and Technology Press; 2008.

[41] Briggs DEG. The role of decay and mineralization in the preservation of soft-bodied fossils. Annu Rev Earth Pl Sc. 2003;31:275–301.

[42] Butterfield NJ. *Leanchoilia* guts and the interpretation of three-dimensional structures in Burgess Shale-type Fossils. Paleobiology. 2002;28(1):155–171.

[43] Butterfield NJ, Balthasar U, Wilson LA. Fossil diagenesis in the Burgess Shale. Palaeontology. 2007;50:537–543.

[44] Gaines RR, Briggs DEG, Zhao Y. Cambrian Burgess Shale-type deposits share a common mode of fossilization. Geology. 2008;36(10):755–758.

[45] Zhu M, Babcock LE, Steiner M. Fossilization modes in the Chengjiang Lagerstätte (Cambrian of China): testing the roles of organic preservation and diagenetic alteration in exceptional preservation. Palaeogeogr Palaeoclimatol Palaeoecol. 2005;220(1–2):31–46.

[46] Forchielli A, Steiner M, Kasbohm J, Hu S, Keupp H. Taphonomic traits of clay-hosted early Cambrian Burgess Shale-type fossil Lagerstätten in South China. Palaeogeogr Palaeoclimatol Palaeoecol. 2014;398:59–85.

[47] Forchielli A, Steiner M, Hu S, Keupp H. Taphonomy of Cambrian (Stage 3/4) sponges from Yunnan (South China). Bull Geosci. 2012;87(1):133–142.

[48] Gabbott SE, Xian-guang H, Norry MJ, Siveter DJ. Preservation of Early Cambrian animals of the Chengjiang biota. Geology. 2004;32(10):901–904.

[49] Luo H, Hu SX, Chen LZ, Zhang SS, Tao YH. Early Cambrian Chengjiang Fauna from Kunming Region, China. Kunming: Yunnan Science and Technology Press; 1999.

[50] Hu S, Zhang Z, Holmer LE, Skovsted CB. Soft-part preservation in a linguliform brachiopod from the lower Cambrian Wulongqing Formation (Guanshan Fauna) of Yunnan, South China. 2010;55(3):495–505.

[51] Daley AC, Drage HB. The fossil record of ecdysis, and trends in the moulting behaviour of trilobites. Arthropod Struct Dev. 2015;45(2):71–96. doi: 10.1016/j.asd.2015.09.004 26431634 10.1016/j.asd.2015.09.004

[52] Ebbestad JOR, Rushton AWA, Stein M, Weidner T. A paradoxidid moult ensemble from the Cambrian of Sweden. GFF. 2013;135(1):18–29.

[53] Ortega-Hernández J, Brena C. Ancestral patterning of tergite formation in a centipede suggests derived mode of trunk segmentation in trilobites. PLoS ONE. 2012;7(12):e52623 doi: 10.1371/journal.pone.0052623 23285116 10.1371/journal.pone.0052623 PMC3532300

[54] Lin J-P. Taphonomy of Naraoiids (Arthropoda) from the Middle Cambrian Kaili Biota, Guizhou Province, South China. Palaios. 2006;21(1):15–25.

