# Supplementary material for: The oldest known digestive system consisting of both paired digestive glands and a crop from exceptionally preserved trilobites of the Guanshan Biota (Early Cambrian, China)
Source: PLoS One. 2017 Sep 21;12(9):e0184982. doi: 10.1371/journal.pone.0184982 (PMC5608306; doi:10.1371/journal.pone.0184982)
Supplement: S1 File — Supporting figures A-Q. (PDF) [file pone.0184982.s001.pdf]

S1 File: Supporting figures A-Q

Associated with: Hopkins, M.J., Chen, F., Hu, S., and Zhang, Z. The oldest known digestive system consisting of both paired digestive glands and a crop from exceptionally preserved trilobites of the Guanshan Biota (Early Cambrian, China). PLOSone.

This document contains:

Fig A. Frequency of preservation of digestive system in Guanshan trilobites. Slab showing specimens of *Palaeolenus lantenoisi*, with white arrows indicating specimens where crop is preserved. Scale bar = 1 cm.

Fig B. Elemental maps of crop of *Palaeolenus lantenoisi*, GLF WLQ 228A (see also Fig 2). Upper left corner: all detected elements. Remaining panels: individual elements of higher concentrations, as labeled. Scale bar as in upper left corner.

Fig C. Elemental maps of digestive glands of *Palaeolenus lantenoisi*, GLF WLQ 228A (see also Fig 2). Upper left corner: all detected elements. Remaining panels: individual elements of higher concentrations, as labeled. Scale bar as in upper left corner.

Fig D. Elemental maps of close-up of digestive glands of *Palaeolenus lantenoisi*, GLF WLQ 228A (see also Fig 2). Upper left corner: all detected elements. Remaining panels: individual elements of higher concentrations, as labeled. Scale bar as in upper left corner.

Fig E. Elemental maps of mid-thoracic segments of *Palaeolenus lantenoisi*, GLF WLQ 228A (see also Fig 2). Upper left corner: all detected elements. Remaining panels: individual elements of higher concentrations, as labeled. Scale bar as in upper left corner.

Fig F. Elemental maps of pygidium of *Palaeolenus lantenoisi*, GLF WLQ 228A (see also Fig 2). Upper left corner: all detected elements. Remaining panels: individual elements of higher concentrations, as labeled. Scale bar as in upper left corner.

Fig G. Elemental maps of red staining posterior to pygidium of *Palaeolenlus lantenoisi*, GLF WLQ 228A (see also Fig 2). Upper left corner: all detected elements. Remaining panels: individual elements of higher concentrations, as labeled. Scale bar as in upper left corner.

Fig H. Elemental maps of cranidium of *Palaeolenus lantenoisi*, GLF WLQ 214A (see also Fig 3E-4F). Upper left corner: all detected elements. Remaining panels: individual elements of higher concentrations, as labeled. Scale bar as in upper left corner.

Fig I. Elemental maps of crop of *Palaeolenus lantenoisi*, GLF WLQ 174 (see also Figs 3A-3D). Upper left corner: all detected elements. Remaining panels: individual elements of higher concentrations, as labeled. Scale bar as in upper left corner.

Fig J. Elemental maps of cranidium of *Palaeolenus lantenoisi*, GLF WLQ 212A (see also Figs 3G and 3I). Upper left corner: all detected elements. Remaining panels: individual elements of higher concentrations, as labeled. Scale bar as in upper left corner.

Fig K. Elemental maps of thorax of *Palaeolenus lantenoisi*, GLF WLQ 212A (see also Figs 3H-3I). Upper left corner: all detected elements. Remaining panels: individual elements of higher concentrations, as labeled. Scale bar as in upper left corner.

Fig L. Elemental maps of crop of *Redlichia mansuyi*, GLF WLQ 245A (see also Figs 3J-3K). Upper left corner: all detected elements. Remaining panels: individual elements of higher concentrations, as labeled. Scale bar as in upper left corner.

Fig M. Elemental maps of left half of cranidium of *Redlichia mansuyi?*, GLF WLQ 029A. Upper left corner: all detected elements. Remaining panels: individual elements of higher concentrations, as labeled. Scale bar as in upper left corner.

Fig N. Elemental maps of thoracic pleural spine of *Redlichia sp.*, GLF WLQ 244A. Upper left corner: all detected elements. Remaining panels: individual elements of higher concentrations, as labeled. Scale bar as in upper left corner.

Fig O. Elemental maps of crop of *Redlichia mansuyi*, GLF WLQ 216A (see also Fig 4). Upper left corner: all detected elements. Remaining panels: individual elements of higher concentrations, as labeled. Scale bar as in upper left corner.

Fig P. Elemental maps of the spherical aggregates (close up) in crop of *Redlichia mansuyi*, GLF WLQ 216A (see also Fig 4). Upper left corner: all detected elements. Remaining panels: individual elements of higher concentrations, as labeled. Scale bar as in upper left corner.

Fig Q. EDS spectrum of spherical aggregates in crop of *Redlichia mansuyi*, GLF WLQ 216A (area shown in Figs 4E-4G and Fig P).

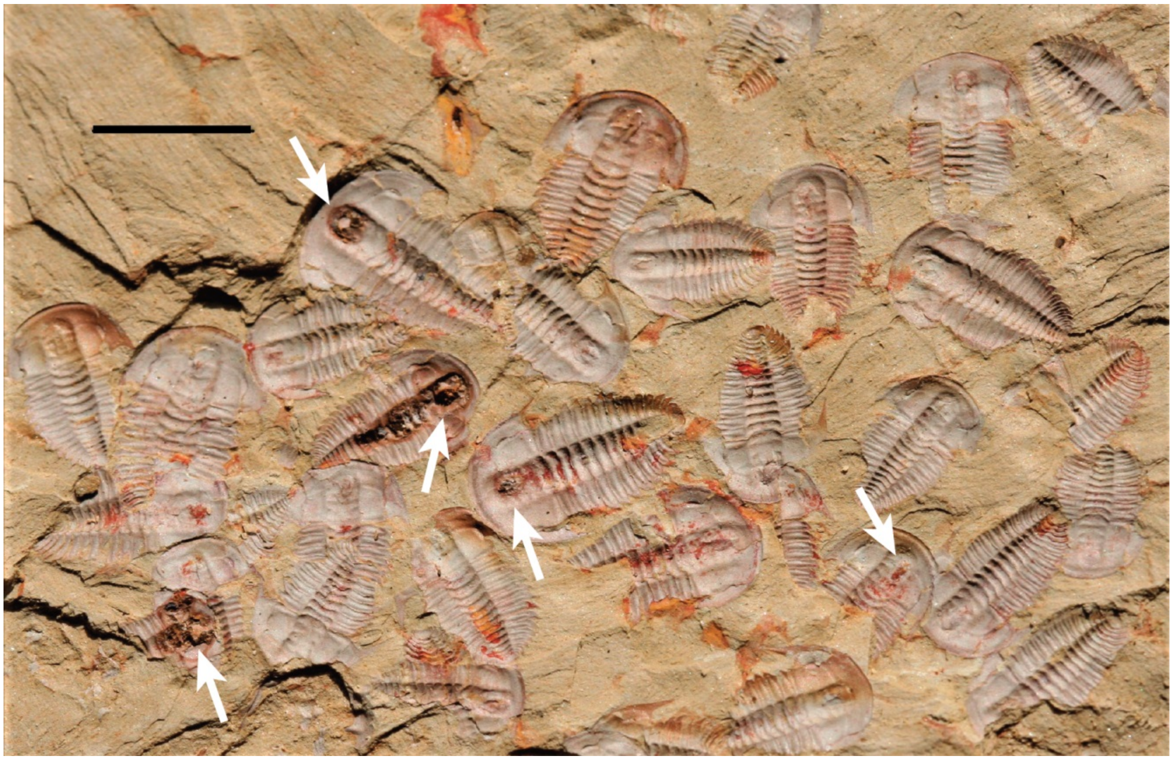

Fig A. Frequency of preservation of digestive system in Guanshan trilobites. Slab showing specimens of *Palaeolenus lantenoisi*, with white arrows indicating specimens where crop is preserved. Scale bar = 1 cm.

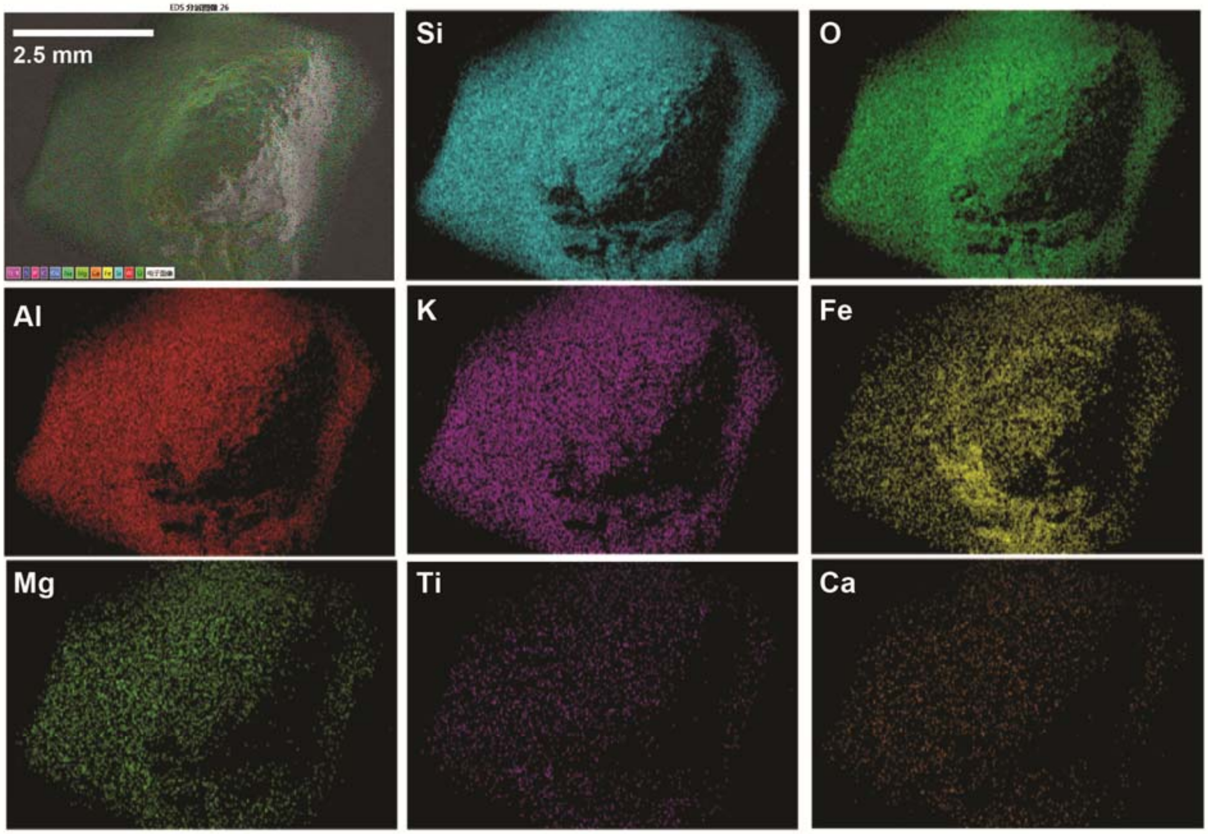

Fig B. Elemental maps of crop of *Palaeolenus lantenoisi*, GLF WLQ 228A (see also Figure 2). Upper left corner: all detected elements. Remaining panels: individual elements of higher concentrations, as labeled. Scale bar as in upper left corner.

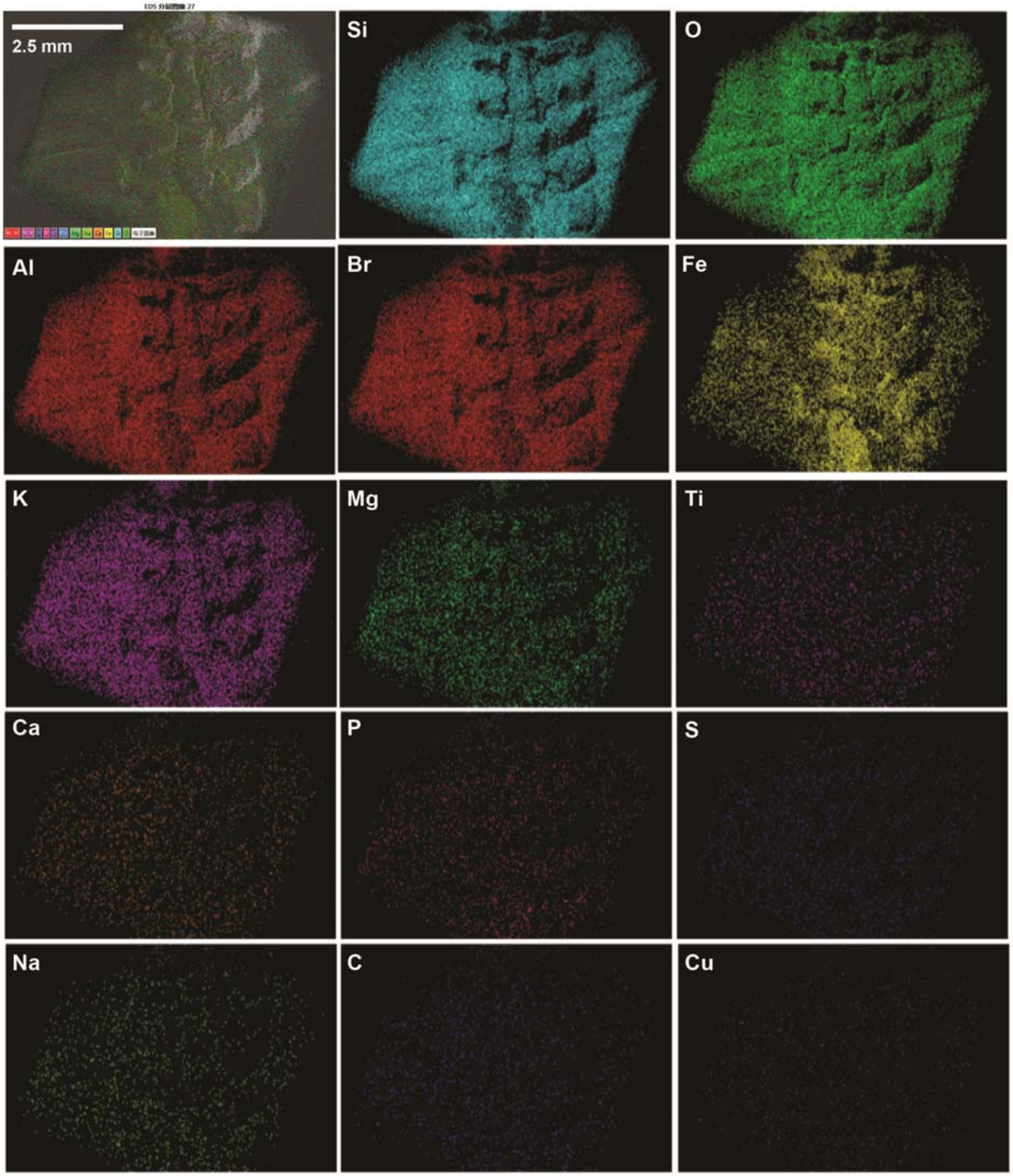

Fig C. Elemental maps of digestive glands of *Palaeolenus lantenoisi*, GLF WLQ 228A (see also Figure 2). Upper left corner: all detected elements. Remaining panels: individual elements of higher concentrations, as labeled. Scale bar as in upper left corner.

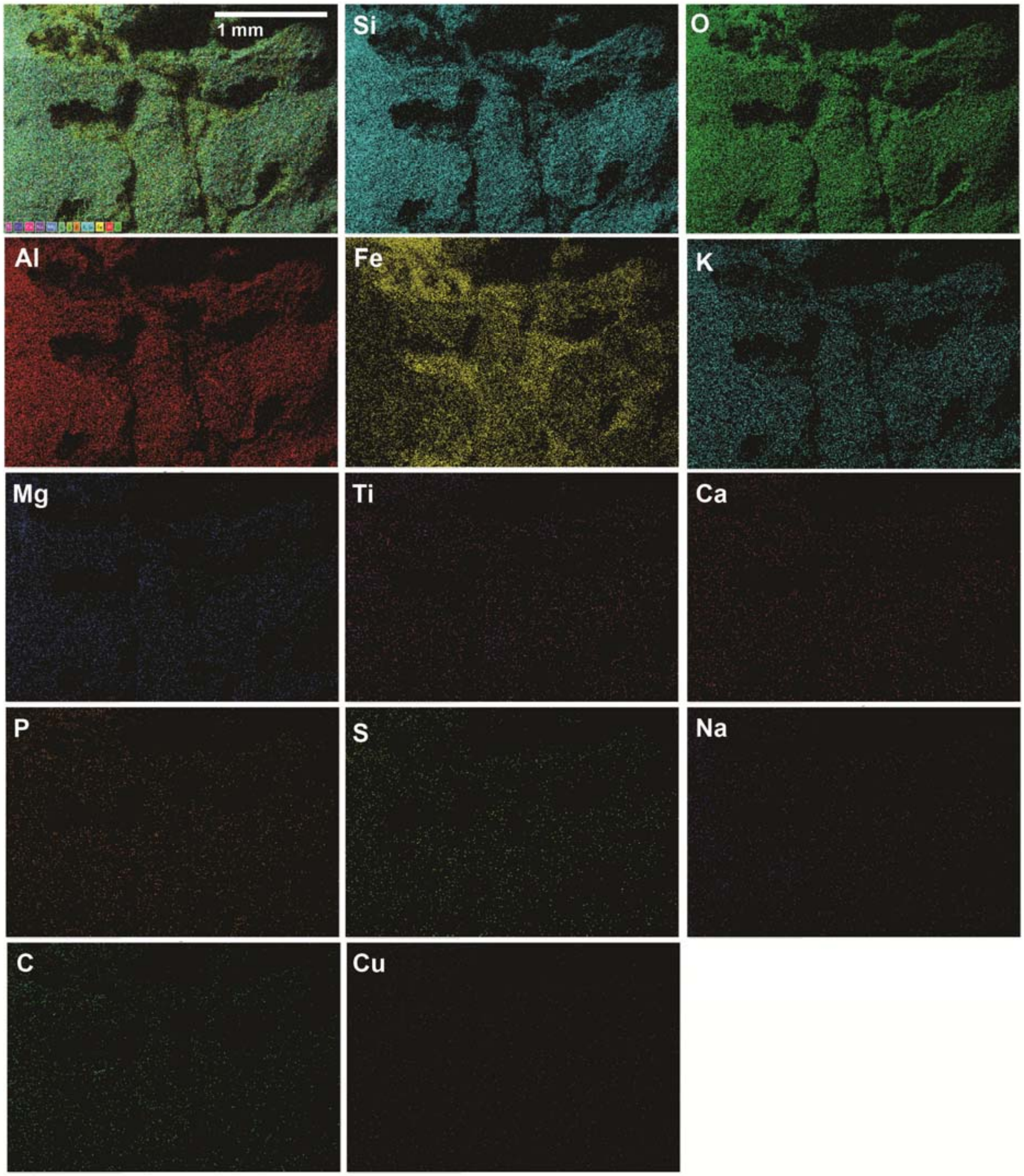

Fig D. Elemental maps of close-up of digestive glands of *Palaeolenus lantenoisi*, GLF WLQ 228A (see also Figure 2). Upper left corner: all detected elements. Remaining panels: individual elements of higher concentrations, as labeled. Scale bar as in upper left corner.

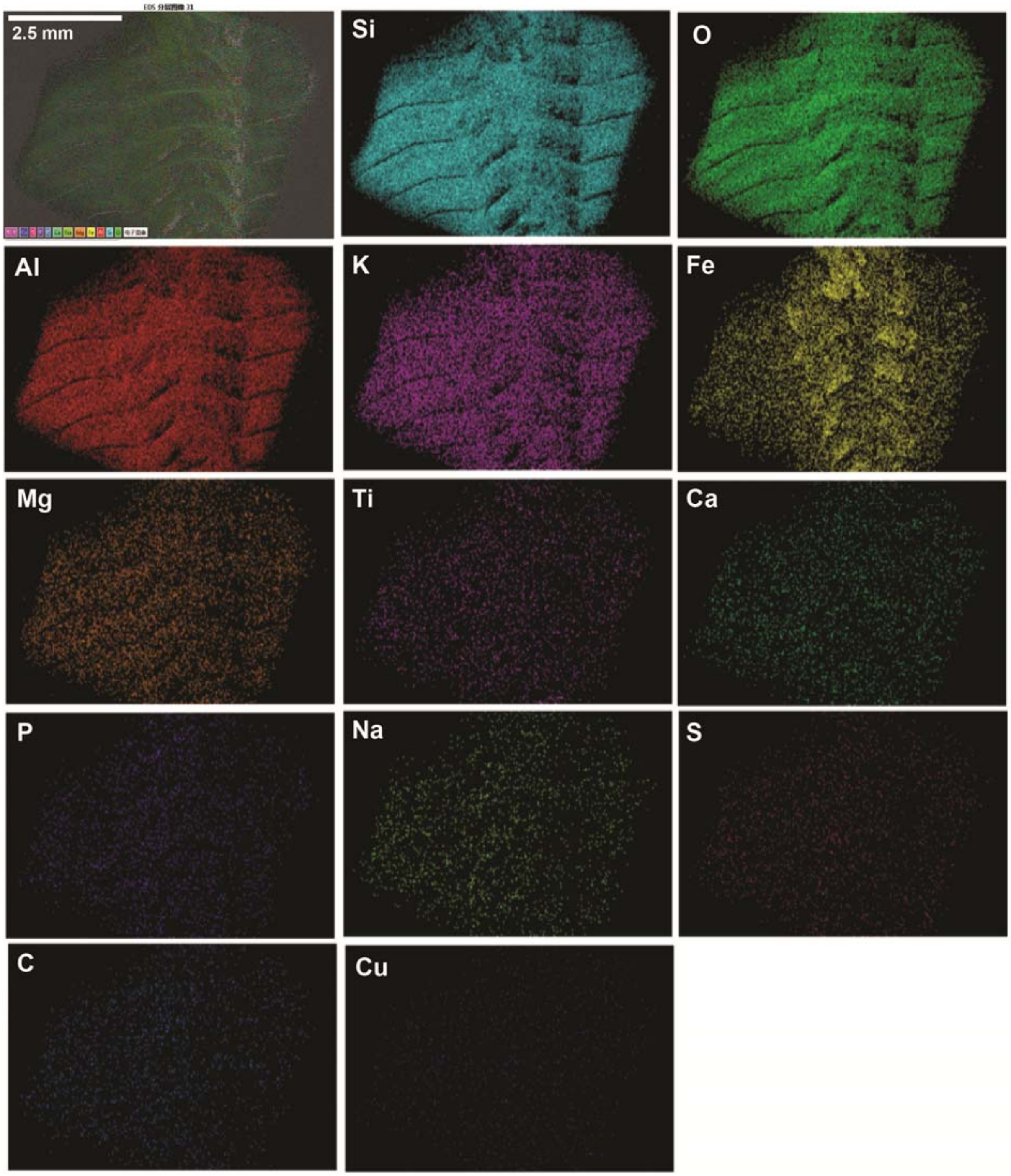

Fig E. Elemental maps of mid-thoracic segments of *Palaeolenus lantenoisi*, GLF WLQ 228A (see also Figure 2). Upper left corner: all detected elements. Remaining panels: individual elements of higher concentrations, as labeled. Scale bar as in upper left corner.

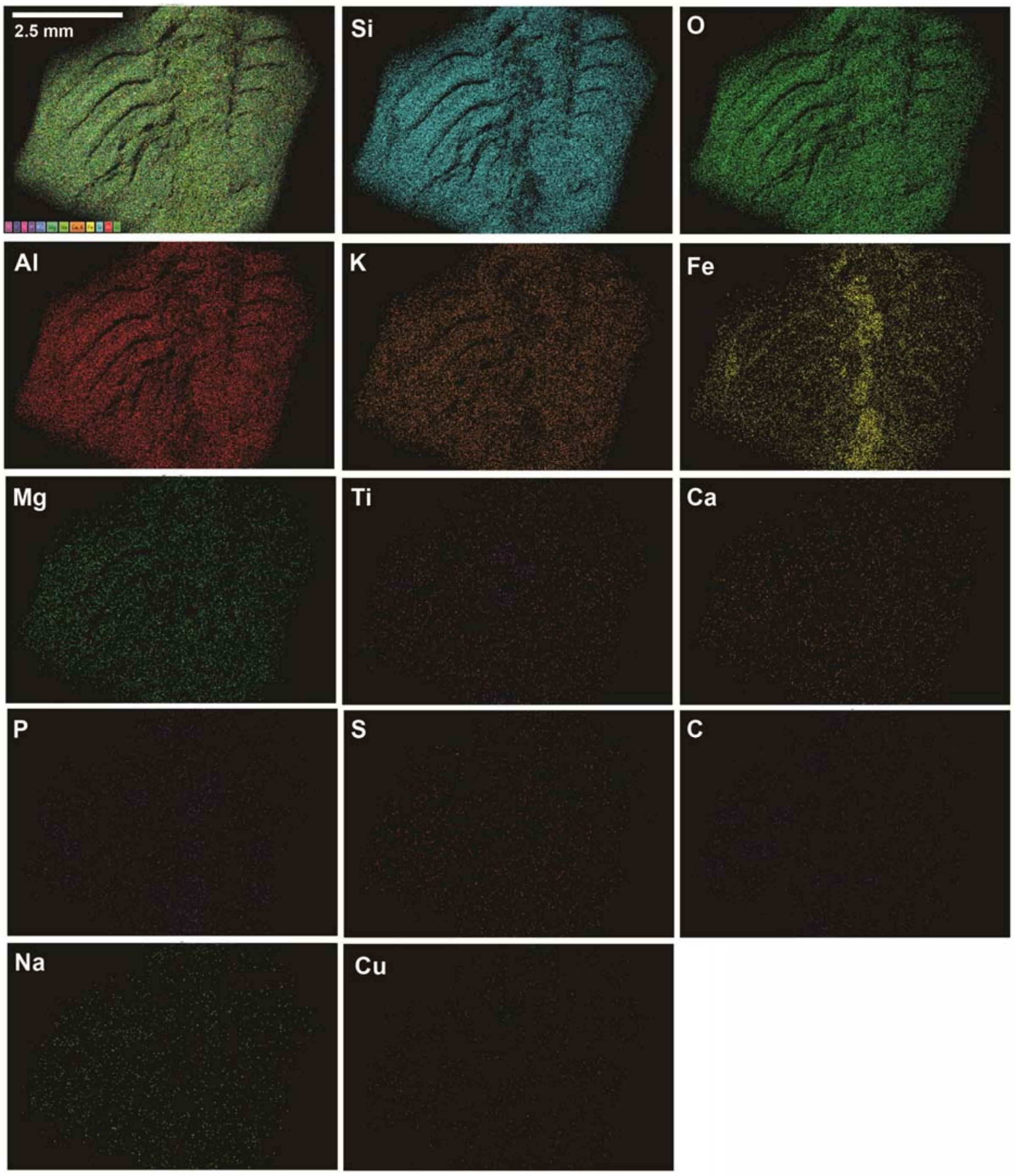

Fig F. Elemental maps of pygidium of *Palaeolenus lantenoisi*, GLF WLQ 228A (see also Figure 2). Upper left corner: all detected elements. Remaining panels: individual elements of higher concentrations, as labeled. Scale bar as in upper left corner.

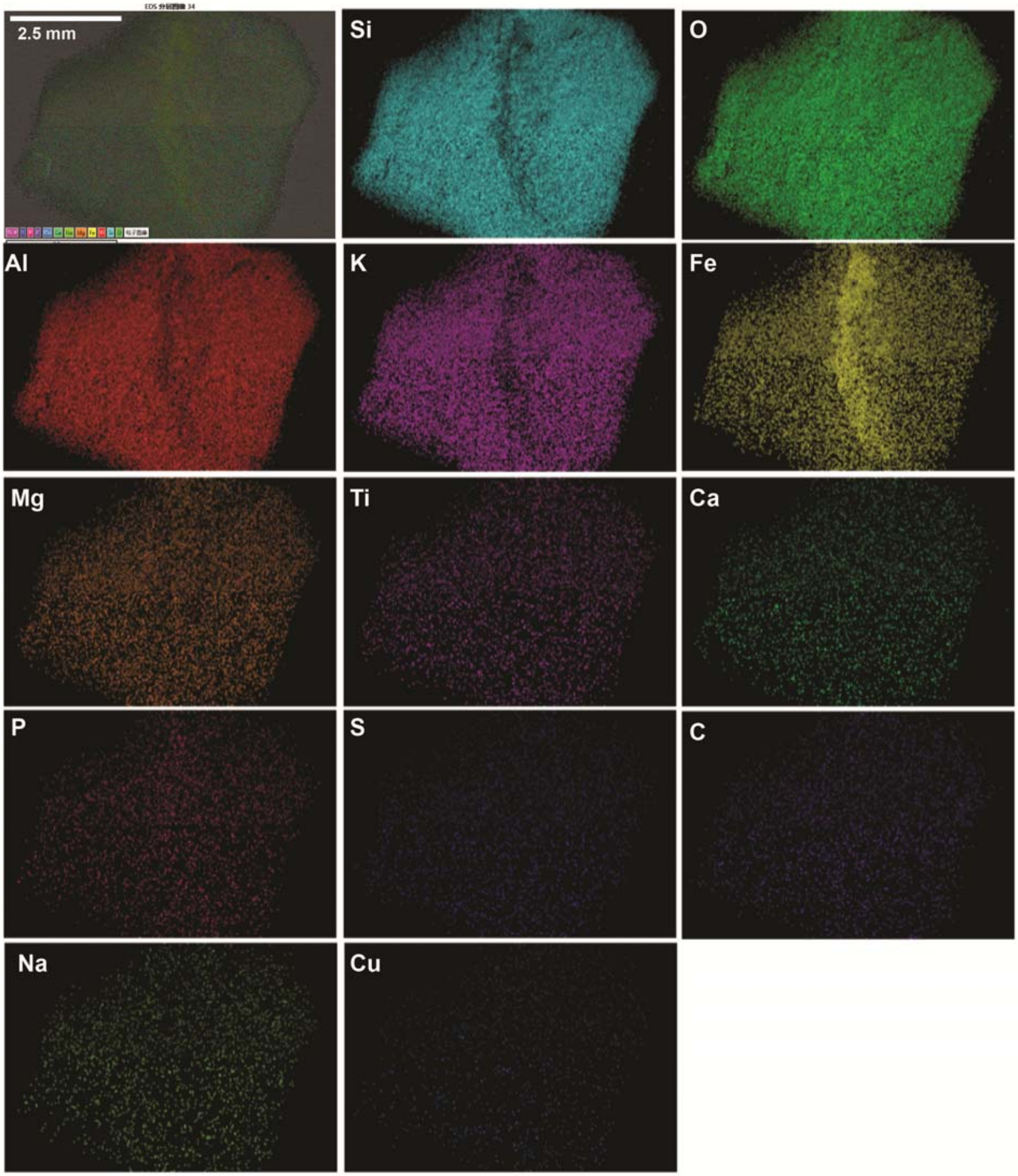

Fig G. Elemental maps of red staining posterior to pygidium of *Palaeolenlus lantenoisi*, GLF WLQ 228A (see also Figure 2). Upper left corner: all detected elements. Remaining panels: individual elements of higher concentrations, as labeled. Scale bar as in upper left corner.

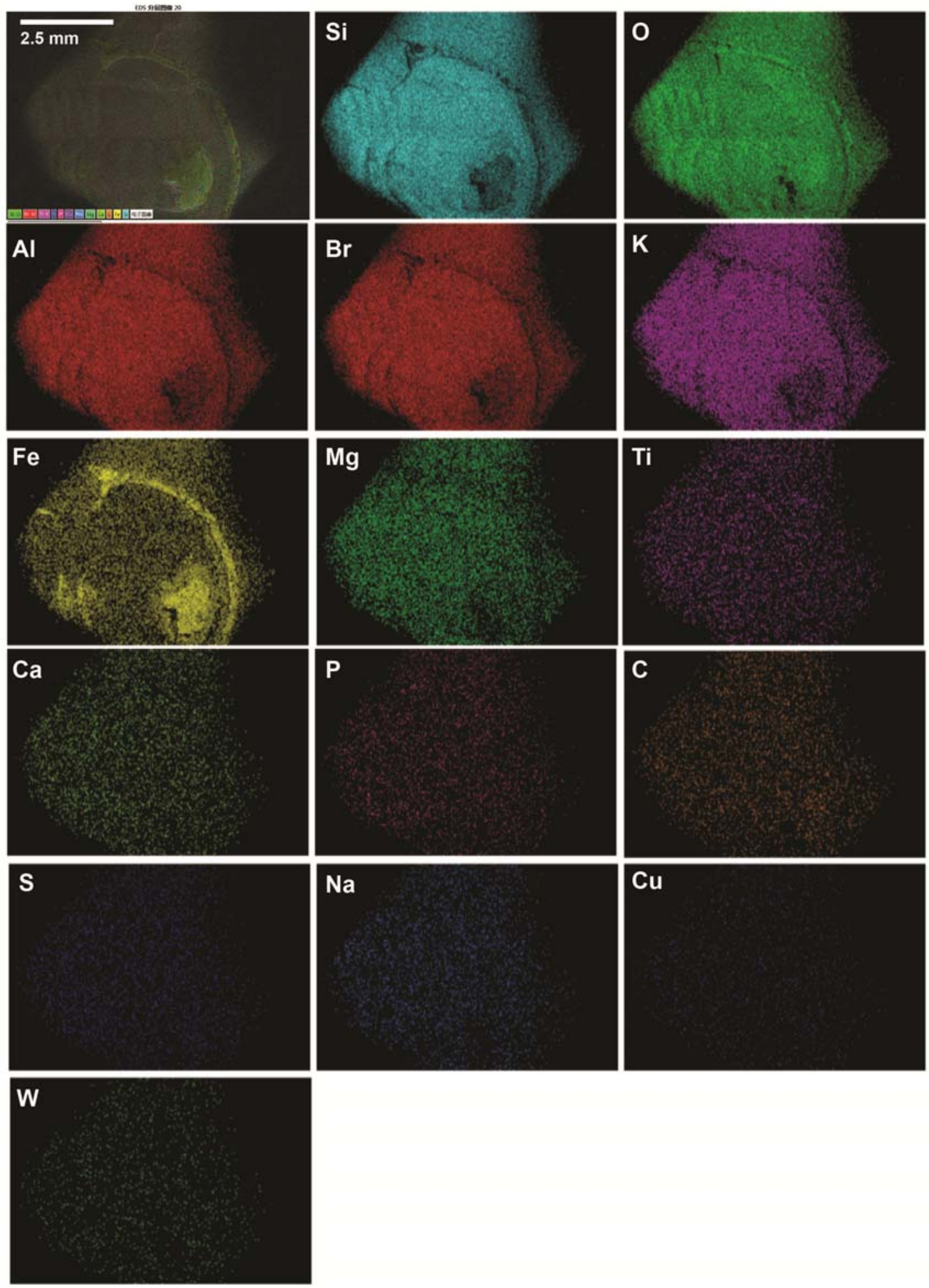

Fig H. Elemental maps of cranidium of *Palaeolenus lantenoisi*, GLF WLQ 214A (see also Figure 3E-3F). Upper left corner: all detected elements. Remaining panels: individual elements of higher concentrations, as labeled. Scale bar as in upper left corner.

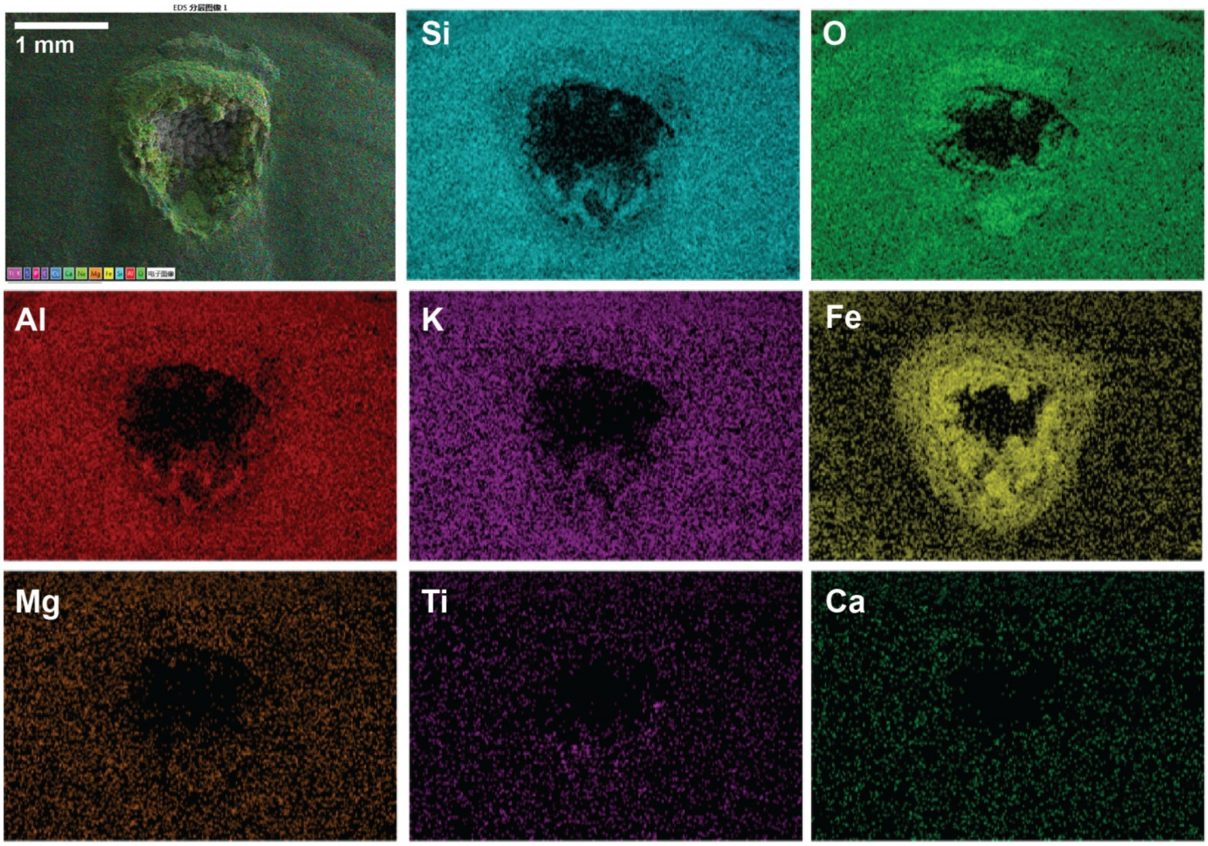

Fig I. Elemental maps of crop of *Palaeolenus lantenoisi*, GLF WLQ 174 (see also Figure 3A-3D). Upper left corner: all detected elements. Remaining panels: individual elements of higher concentrations, as labeled. Scale bar as in upper left corner.

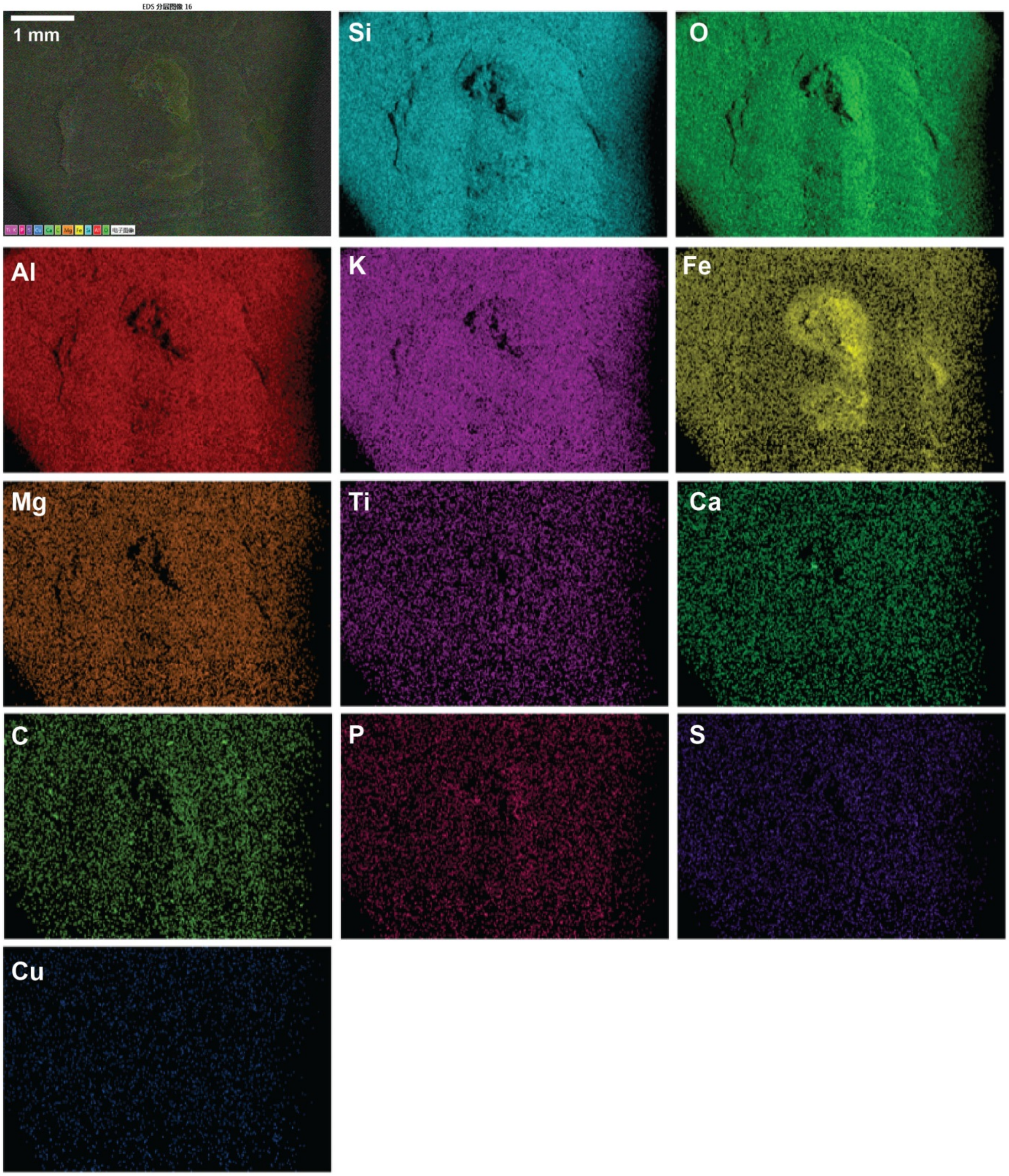

Fig J. Elemental maps of cranidium of *Palaeolenus lantenoisi*, GLF WLQ 212A (see also Figure 3G, 3I). Upper left corner: all detected elements. Remaining panels: individual elements of higher concentrations, as labeled. Scale bar as in upper left corner.

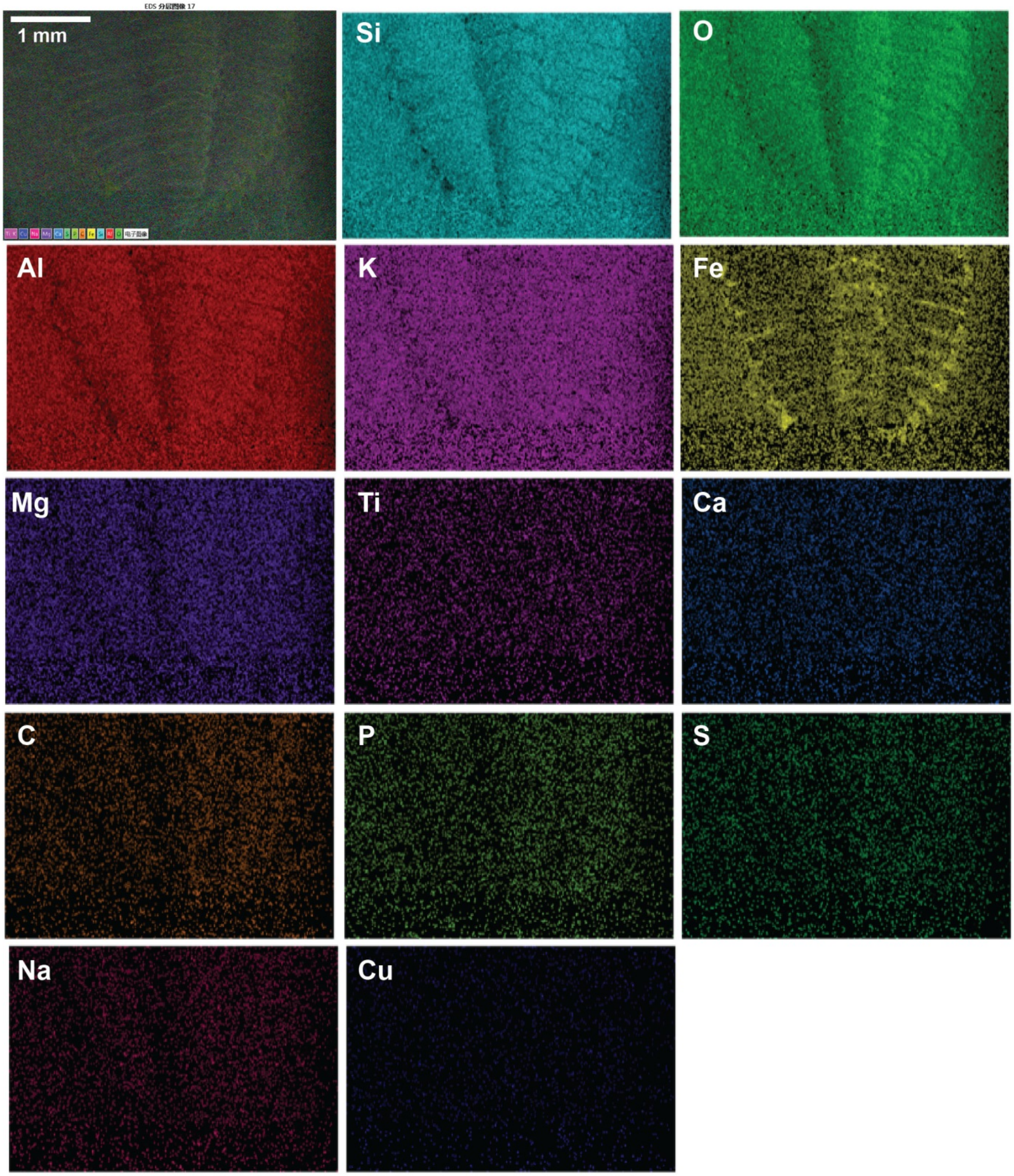

Fig K. Elemental maps of thorax of *Palaeolenus lantenoisi*, GLF WLQ 212A (see also Figure 3H-3I). Upper left corner: all detected elements. Remaining panels: individual elements of higher concentrations, as labeled. Scale bar as in upper left corner.

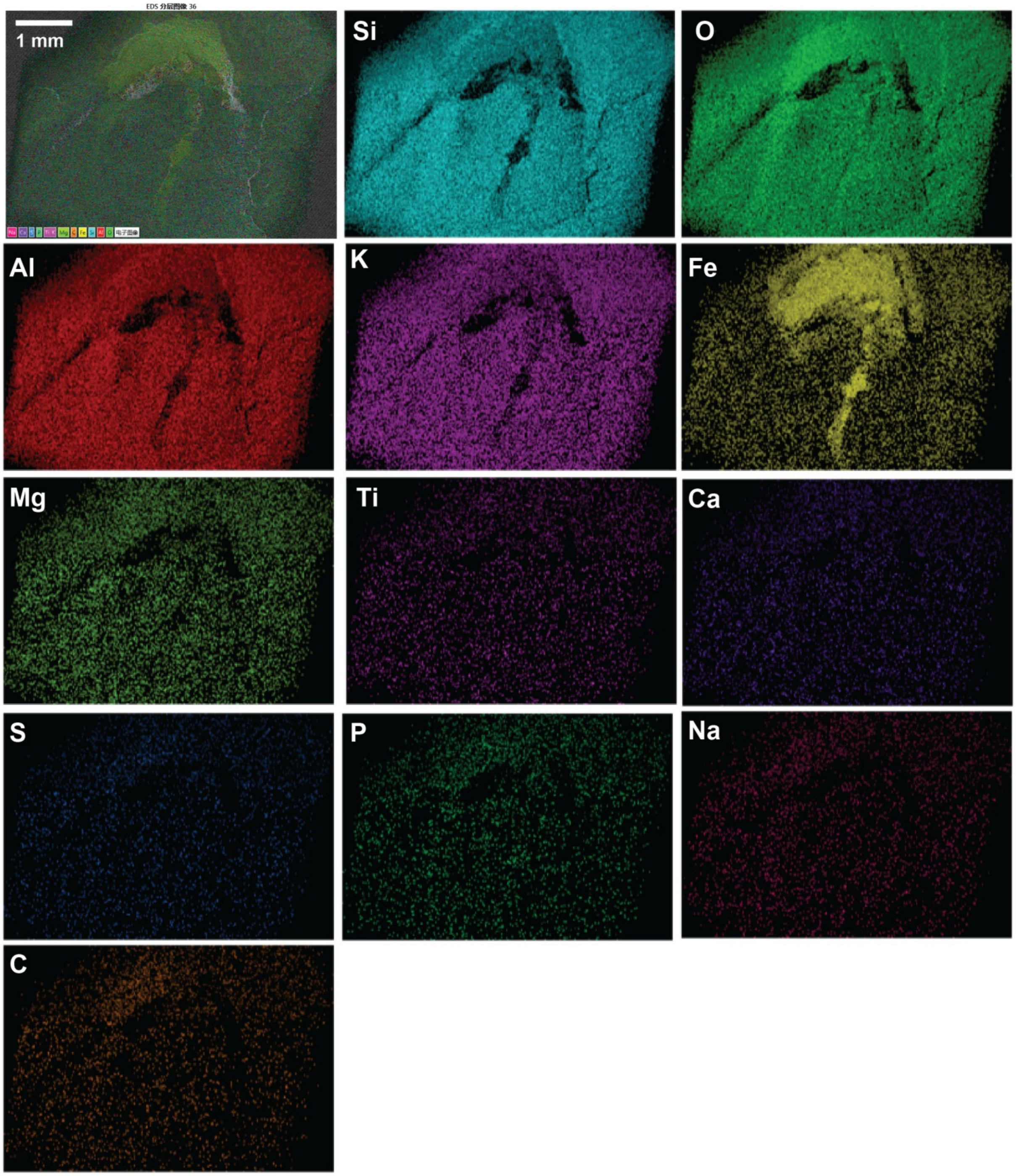

Fig L. Elemental maps of crop of *Redlichia mansuyi*, GLF WLQ 245A (see also Figure 3J-3K). Upper left corner: all detected elements. Remaining panels: individual elements of higher concentrations, as labeled. Scale bar as in upper left corner.

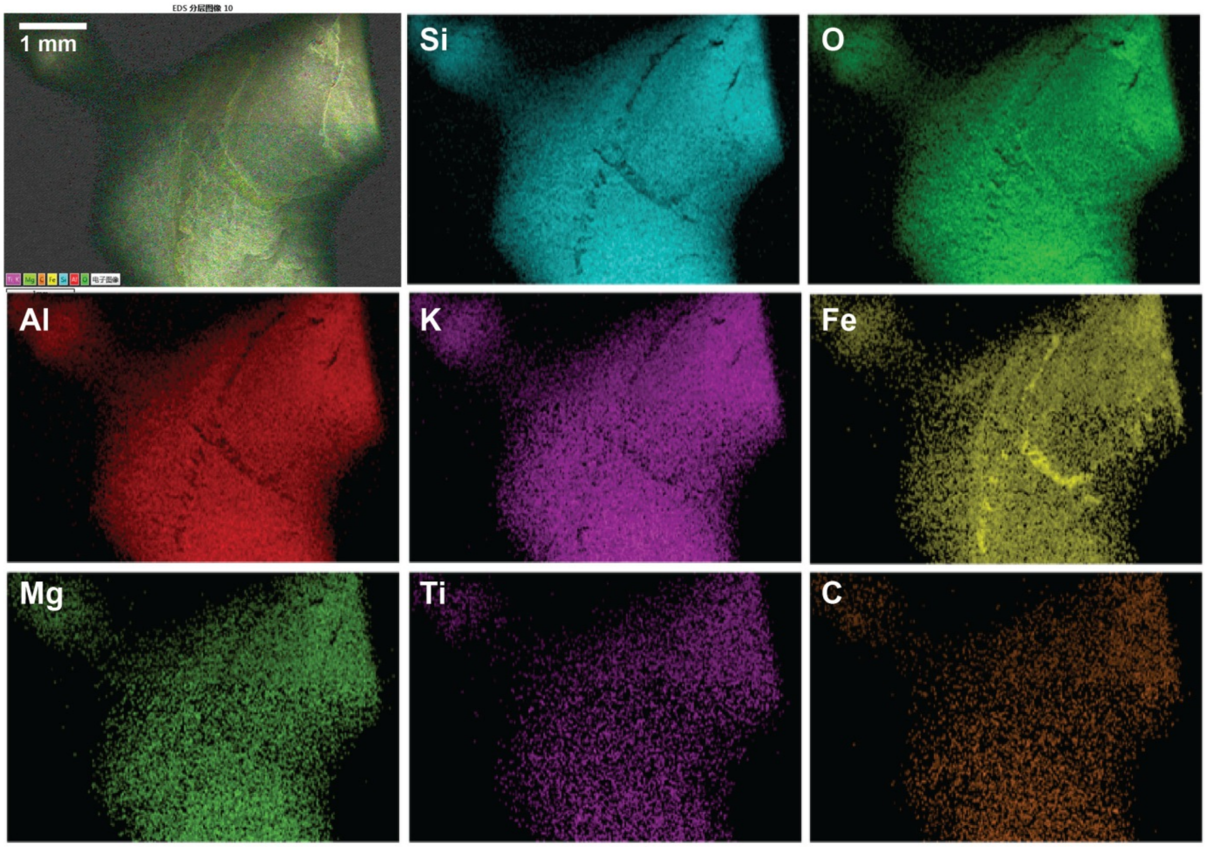

Fig M. Elemental maps of left half of cranidium of *Redlichia mansuyi?*, GLF WLQ 029A. Upper left corner: all detected elements. Remaining panels: individual elements of higher concentrations, as labeled. Scale bar as in upper left corner.

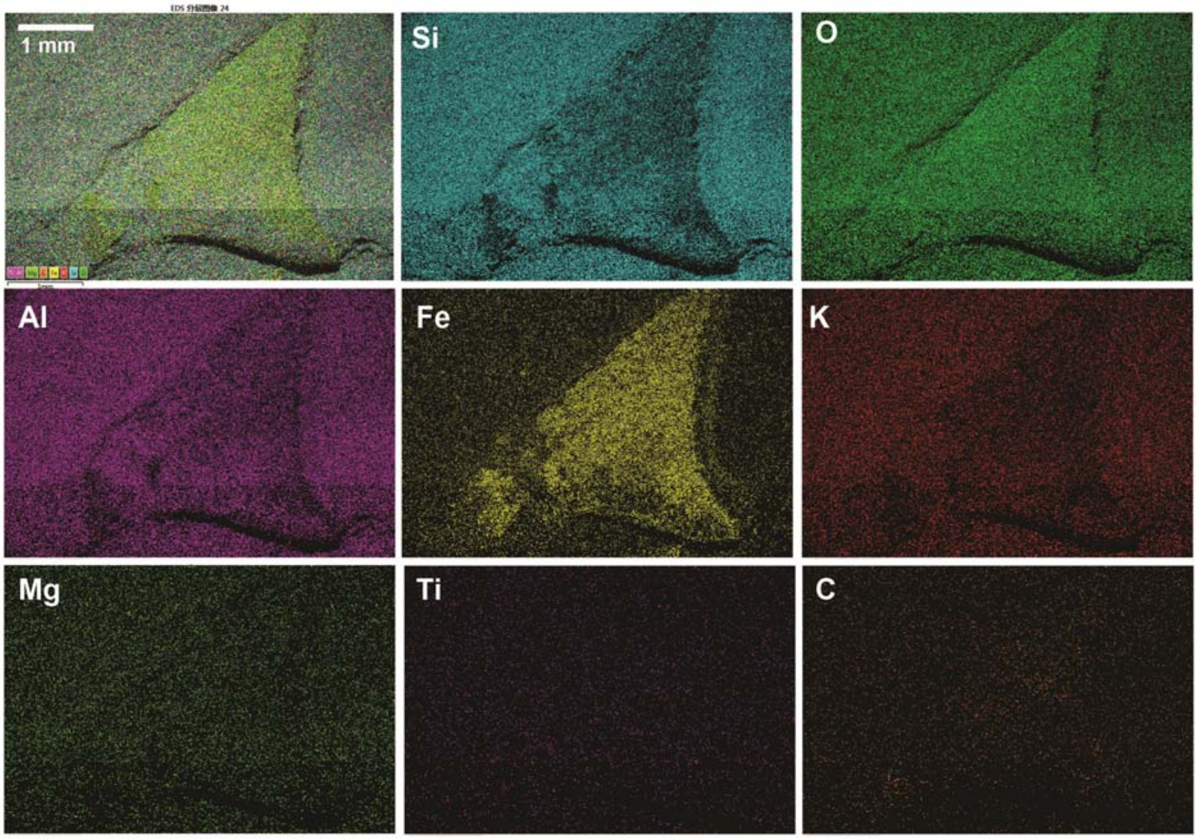

Fig N. Elemental maps of thoracic pleural spine of *Redlichia sp*., GLF WLQ 244A. Upper left corner: all detected elements. Remaining panels: individual elements of higher concentrations, as labeled. Scale bar as in upper left corner.

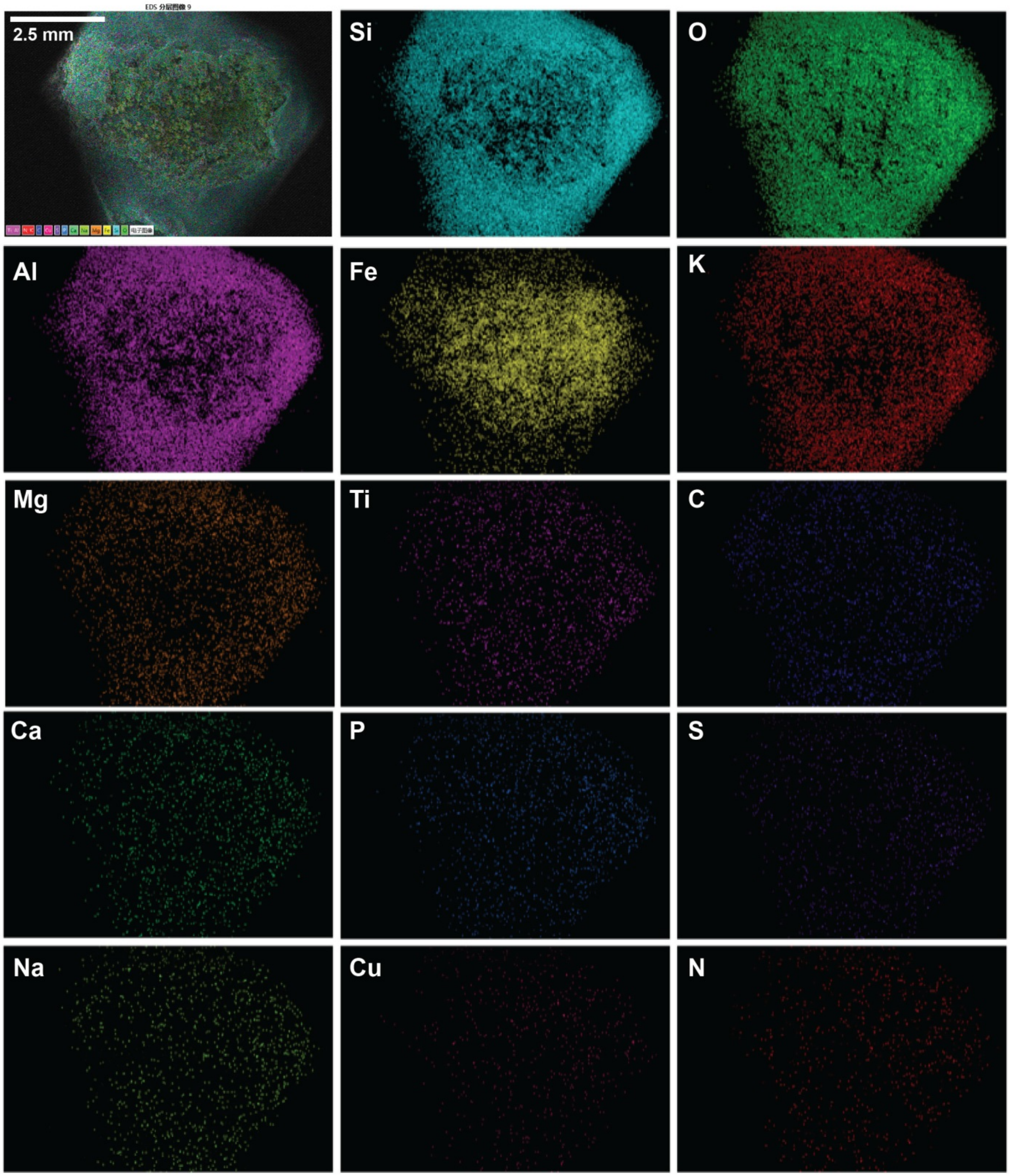

Fig O. Elemental maps of crop of *Redlichia mansuyi*, GLF WLQ 216A (see also Figure 4). Upper left corner: all detected elements. Remaining panels: individual elements of higher concentrations, as labeled. Scale bar as in upper left corner.

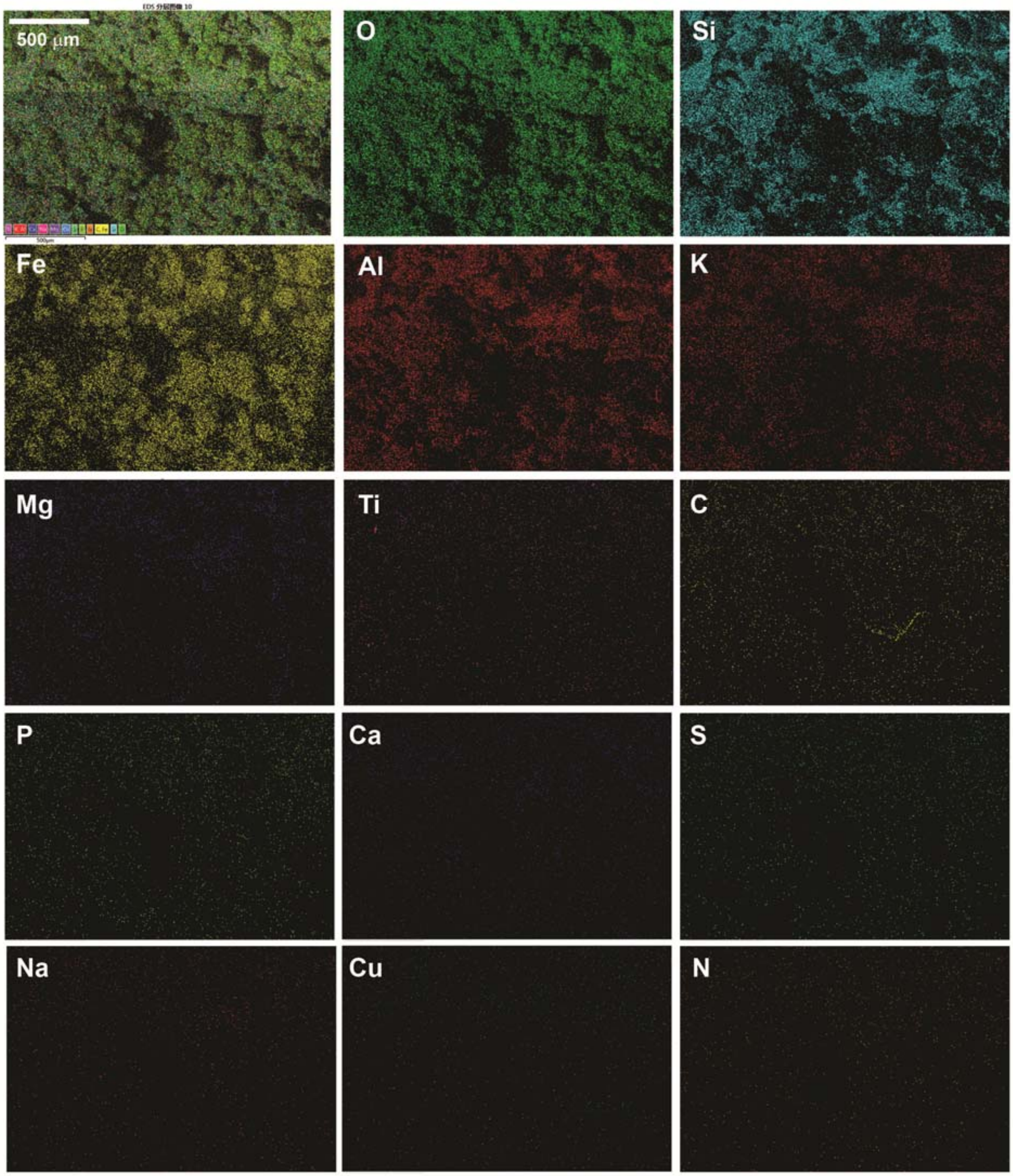

Fig P. Elemental maps of spherical aggregates in crop of *Redlichia mansuyi*, GLF WLQ 216A (see also Figure 4). Upper left corner: all detected elements. Remaining panels: individual elements of higher concentrations, as labeled. Scale bar as in upper left corner.

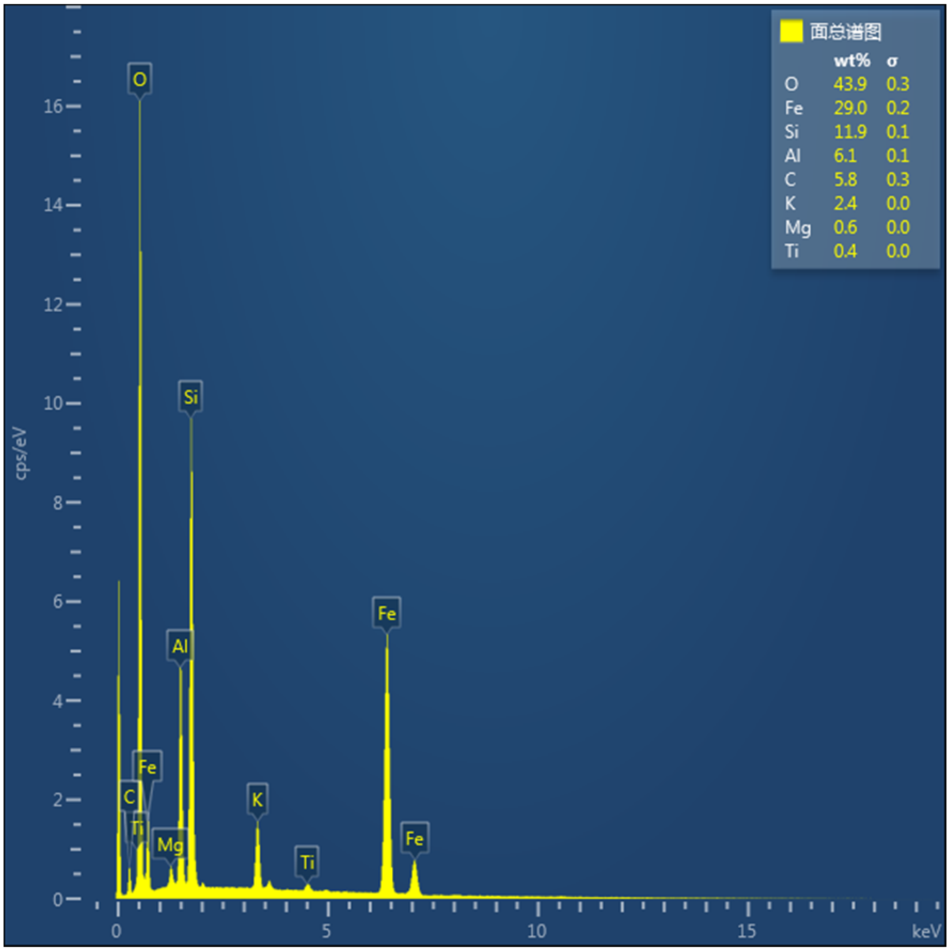

Fig Q. EDS spectrum of spherical aggregates in crop of *Redlichia mansuyi*, GLF WLQ 216A (area shown in Figure 4E-G and Figure S16).
